# Determinants of participation in voluntary work: a systematic review and meta-analysis of longitudinal cohort studies

**DOI:** 10.1186/s12889-018-6077-2

**Published:** 2018-11-02

**Authors:** Jacobien Niebuur, Lidy van Lente, Aart C. Liefbroer, Nardi Steverink, Nynke Smidt

**Affiliations:** 1 Department of Epidemiology, University of Groningen, University Medical Center Groningen, Hanzeplein 1, PO Box 30 001, FA40, 9700 RB Groningen, The Netherlands; 2 Netherlands Interdisciplinary Demographic Institute, The Hague, the Netherlands; 3 Department of Sociology, Vrije Universiteit Amsterdam, Amsterdam, the Netherlands; 4 Department of Sociology, University of Groningen, Groningen, the Netherlands; 5 Department of Health Psychology, University of Groningen, University Medical Center Groningen, Groningen, the Netherlands; 6 Department of Geriatrics, University of Groningen, University Medical Center Groningen, Groningen, The Netherlands

**Keywords:** Social participation, Volunteers, Factors, Determinants, Participation, General population, Unpaid work, Review, Meta-analysis

## Abstract

**Background:**

Participation in voluntary work may be associated with individual and societal benefits. Because of these benefits and as a result of challenges faced by governments related to population ageing, voluntary work becomes more important for society, and policy measures are aimed at increasing participation rates. In order to effectively identify potential volunteers, insight in the determinants of volunteering is needed. Therefore, we conducted a systematic review including meta-analyses.

**Methods:**

A systematic search in MEDLINE, PsycINFO, SocINDEX, Business Source Premier, and EconLit was performed on August 12th 2015. We included longitudinal cohort studies conducted in developed countries that quantified factors associated with volunteering among samples from the general adult population. Two reviewers independently selected eligible studies, extracted the data and assessed the risk of bias of the included studies using the QUIPS tool. Estimates reported in the papers were transformed into Odds Ratios and 95% Confidence Intervals. For each determinant, random-effects meta-analyses were used to generate summary estimates.

**Results:**

We found that socioeconomic status, being married, social network size, church attendance and previous volunteer experiences are positively associated with volunteering. Age, functional limitations and transitions into parenthood were found to be inversely related to volunteering.

**Conclusions:**

Important key factors have been identified as well as gaps in the current literature. Future research should be directed towards deepening the knowledge on the associations between the factors age, education, income, employment and participation in voluntary work. Moreover, major life course transitions should be studied in relation to volunteering.

## Background

Participation in voluntary work can have several individual and societal benefits. It is inversely related to mortality [1, 2] depression [2, 3] and functional limitations [3], and positively related to self-rated health [3]. In turn, improved individual health is reflected in more societal sustainability, for example in terms of health care systems [4]. Furthermore, societal benefits of volunteering include increases in social solidarity and individuals’ involvement in society [5] as well as economic benefits, for example in terms of contributions to Gross Domestic Product levels [6]. Because of the various socioeconomic benefits of volunteering and because of the current challenges faced by many developed countries related to population ageing, many policy measures are aimed nowadays at increasing participation rates in volunteering. In order to effectively target potential volunteers and to utilize the benefits related to volunteering, there is a need to understand the key factors related to participation in voluntary work. One important set of key factors are socio-demographic characteristics. By socio-demographic characteristics we mean characteristics that signify an individual’s position in society. This includes indicators of an individual’s position in the family domain (such as partner status and social network integration), the economic domain (such as education and income) and in the health domain (such as wellbeing). All these socio-demographic characteristics are examples of factors for which an association with volunteering is expected. Our research questions are:

1. What are the determinants (e.g. socio-demographic characteristics) of participation in voluntary work?
2. What is the magnitude and direction of the relationship between identified determinants (e.g. socio-demographic characteristics) and participation in voluntary work?

Voluntary work is defined as “unpaid non-compulsory work; that is, time individuals give without pay to activities performed either through an organization or directly for others outside their own household” [5]. Research on factors influencing participation in voluntary work is extensive. However, there is large heterogeneity in the determinants measured as well as in the findings. Inconsistencies in findings may result from, among other factors, the use of incomparable study samples, the use of different study designs and the omission of important confounders in analyses. By conducting a systematic review and meta-analysis, sources of heterogeneity in the findings can be further explored and reliable key factors influencing participation in voluntary work can be identified.

Although earlier systematic reviews on determinants of participation in voluntary work provide important contributions to the knowledge on factors related to volunteering, most of them focussed on study samples consisting exclusively of volunteers recruited at voluntary organizations [7, 8], older people, [7] or volunteers working for a specific cause (i.e. volunteering in the care of people with mental illnesses) [8]. Moreover, both reviews included studies using diverse study designs (both quantitative as well as qualitative), and findings were not quantified [7, 8]. Wilson [9] provided an overview of theories explaining volunteerism and described several well-known determinants of volunteering, including level of education (positive association), age (curvilinear relationship), gender (in North-America, women are more likely to volunteer than men), marital status (married people are more likely to volunteer than non-married people) and health status (positive relationship). As the overview is based on literature published up until the year 2000, the findings did not result from conducting a review following a systematic approach, and associations were not quantified by conducting meta-analyses, there is need for updating the knowledge on the determinants of participation in voluntary work. Our aim was to improve the current knowledge by conducting a systematic review including a meta-analysis. Thereby, we aimed at summarizing the available evidence on the determinants of participation in voluntary work and determining the magnitude and direction of the relationship between identified determinants and participation in voluntary work.

## Methods

This systematic review was conducted according to the methods of the Cochrane Collaboration [10] and reported according to the PRISMA (Preferred Reporting Items for Systematic Reviews and Meta-Analyses) guidelines [11].

### Search strategy and study selection

A search was conducted in MEDLINE, PsychINFO, SocINDEX, Business Source Premier and EconLit, on August 12th, 2015. The search strategy included a combination of terms related to (a) participation in voluntary work (e.g. voluntary work, volunteers, unpaid work) and (b) determinants (e.g. determinant, factor, association, relation, reason) (see Appendix 1).

Articles were selected if they are (a) peer-reviewed full text publications reporting an association between at least one individual factor (contextual factors are beyond the scope of this study) and participation in formal voluntary work (i.e. voluntary work carried out for organizations [12]) (yes/no) in a quantitative way using a longitudinal prospective cohort study design (i.e. studies in which the determinant is measured at a moment in time before the outcome was measured), and (b) making use of a study sample consisting of adults aged 18 and over from a general population from a developed country (i.e. Japan and countries in Europe, North America and Oceania). Moreover, (c) the article has to be published in English, French, German or Dutch within the time period 2010–2015. Given the large number of publications on the topic, we decided to focus on recent publications from 2010 onwards. Articles exclusively including informal volunteering as the outcome were excluded. In case it is unclear whether volunteering was formal or informal, articles were included and labelled as ‘mixed type of voluntary work’. Finally, articles focusing on very specific cases of volunteering such as disaster volunteering, corporate volunteering and volunteer-tourism were excluded as well, because of their limited comparability with volunteering in the general population, but also because the motives to participate in these kinds of voluntary work may differ from situation to situation. We focus on longitudinal rather than on cross-sectional studies, as the former offer better opportunities for temporal ordering of factors.

The titles and abstracts of all identified records were screened for eligibility by two reviewers (J.N. and L.v.L.) independently. Subsequently, the same two reviewers independently screened the full-text of all potentially eligible articles. Finally, all references of included articles were screened by one reviewer (J.N.) for potentially eligible articles.

### Data extraction and assessment of risk of bias

Two reviewers (J.N. and L.v.L.) independently extracted the data regarding the characteristics of the study sample (country, mean age, % female, inclusion criteria), the year of baseline measurement, study duration, determinant measurement, outcome measurement, sample size, volunteering at baseline (%), volunteering at follow-up (%), and the results (association between the determinant(s) and the outcome). The same two reviewers independently assessed the risk of bias of the included articles by using the QUIPS (Quality In Prognosis Studies) tool [13]. The following domains were assessed as potential sources for risk of bias: study participation, study attrition, measurement of the determinants and the outcome, study confounding and statistical analysis and reporting (see Appendix 2). Overall disagreement was evaluated and expressed as percentage of agreement and kappa statistics [14]. In a consensus meeting disagreements were discussed and resolved. If consensus could not be reached, a third reviewer (N.Sm.) made the final decision.

### Statistical analysis

In case the results of at least two studies are available, meta-analyses were conducted, using the statistical program Comprehensive Meta-Analysis (3rd version). If studies present several models, estimates from the most complete (fully adjusted) model were used. Odds Ratios (ORs) with 95% Confidence Intervals (CIs) were used, or if needed calculated using the supplemental material of Kuiper et al. [15], to conduct meta-analyses. When insufficient information was available for transforming effect sizes to ORs with 95% CIs, study authors were contacted to obtain the missing information.

In case articles used the same study sample, a-priori defined criteria were used to select the study for the meta-analysis. In order of importance and for each determinant separately, articles were selected based on (a) outcome used in the study (‘formal voluntary work’ was preferred above ‘mixed type of voluntary work’), (b) measurement of the determinant (the determinant measurement was most comparable to other included studies), (c) study sample (the study sample that was the most comparable to the study samples of included studies in the meta-analysis, in terms of the proportion of volunteers at baseline, the age range of participants at baseline, and inclusion criteria for the baseline study sample), (d) sample size (the study with the largest sample size was preferred over smaller studies), and (e) number of determinants quantitatively measured in the study. In case articles presented both a static (e.g. being married) as well as a change score (e.g. transition into marriage) for a certain determinant, the score that is most comparable to the scores used in other included studies for this determinant was used. A random effect method was applied to calculate pooled effect sizes [10].

### Meta-regression and subgroup analyses

Heterogeneity between studies was assessed by using the Index of Inconsistency (*I*^2^) [16]. In case of substantial heterogeneity (*I*^2^ > 50%), sources of heterogeneity between studies were explored by conducting either subgroup analysis (in case < 10 studies are available) or univariable random-effects meta-regression [10] (in case ≥10 studies are available) with regard to the following a-priori defined criteria: (a) outcome measurement (formal voluntary work versus mixed measure); (b) determinant measurement, based on (b1) measurement scale (continuous versus dichotomous scores), (b2) type of measurement (static versus change scores, because the presence of a certain event, (e.g. being married), may have a different association with the outcome than the transition into a certain event (e.g. transition into marriage)), and (b3) conceptual differences in the measurement of the determinant; (c) proportion of volunteers in the baseline study sample; (d) mean age at baseline, because some determinants may be important to a different extent for study samples for which participation in paid work is more or less common; (e) continent in which the study was performed (United States of America (USA), Europe, other), because differences in government regimes and culture may influence the association between a certain determinant and the outcome; (f) year of baseline measurement, because although the included studies were published between 2010 and 2015, the baseline measurement year varies substantially and determinants of participation in voluntary work may differ for different birth cohorts; (g) duration of follow-up (for time-variant variables only); and (h) the risk of bias for each methodological quality domain separately (low risk of bias versus high/unclear risk of bias).

### Publication bias

The likelihood of publication bias was assessed graphically by constructing funnel plots for each determinant (in case at least ten studies were available) using the statistical program Comprehensive Meta-Analysis (3rd version). Asymmetry of the funnel plots was tested using Egger’s method. Publication bias is likely if *p* < 0.10 [17].

## Results

**Fig. 1:**
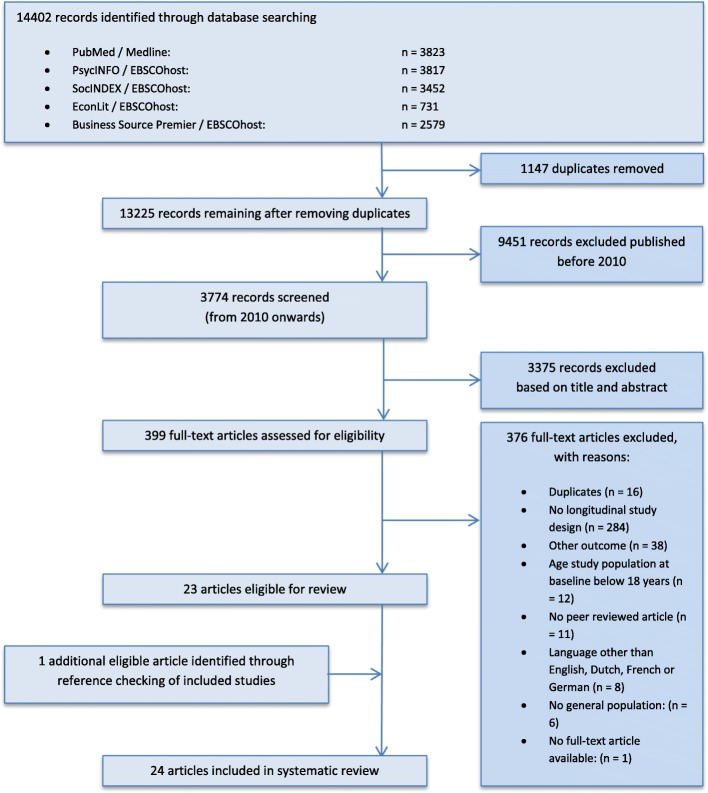
Flow Diagram representing the selection process of articles

The search resulted in the identification of 13.225 records after removing duplicates. A total of 3774 records were published in 2010 or later. The selection process is presented in Fig. 1. Finally, 24 articles were included in the systematic review [18–41]. Characteristics of the included articles are provided in Table 1**.** In Appendix 3 an overview of all determinants measured in included studies is provided.

**Table 1:** Characteristics of included studies

Author	Cohort	Country	Study population^a^	Mean age^b^	SD age^b^	Range age^b^ (years)	Female (%)	Year of baseline^c^	Study duration (years)	Waves (*n*)	Outcome	Outcome measurement	Type of voluntary work^d^	Sample size (n)	Volunteering at baseline (%)	Volunteering at follow-up (%)
Ajrouch et al. [18]	SRHLC^e^	USA^f^	Adults aged ≥50 y	53,9	N.R.^g^	50-100	60,3	1992	13	2	Volunteering {No vs. Yes}	“Do you do any volunteering?”	Mixed	499	N.R.	32,3
Bartels et al. [19]	BHPS^h^	UK^i^	Employed individuals aged ≤60 y	N.R.	N.R.	N.R.	N.R.	1991	16	11	Volunteering {Yes vs. No}	Volunteering is measured as “being active in organizations”	Formal	12,378	N.R.	N.R.
Bekkers [20]	GINPS^j^	NL^k^	N.R.	N.R.	N.R.	N.R.	N.R.	2002	4	3	- Volunteer engagement - Volunteer cessation	Volunteering is measured as “being active as a volunteer in the past year”	Mixed	1233^l^; 731^m^	56,6	44,1
Broese van Groenou & Van Tilburg [21]	LASA^n^	NL	Adults aged between 55 and 69	65,1	5,0	55–69	N.R.	1992^o^/2002^p^	6	3^q^	Volunteering {Yes vs. No}	Current volunteering	Formal	1357^o^; 1388^p^	38,0^o^/45,0^p^	N.R.
Choi & Chou [22]	MIDUS^r^	USA	English speaking adults aged 55–84 y at wave 2 with ≥1 telephone in the household	N.R.	N.R.	N.R.	54,0	1995/1996	9	2	- Volunteer engagement - Volunteer cessation	“On average, about how many hours per month do you spend doing formal volunteer work?”	Formal	917	35,6	41,4
Cramm & Nieboer [23]	N.A.^s^	NL	Older adults aged ≥70 y living in Rotterdam	77,5	5,8	70–101	57,0	2011	2	2	Volunteering {Yes vs. No}	Voluntary activities carried out in the past year	Formal	588	18,5	15,5
Curl et al. [24]	HRS^t^	USA	Adults aged ≥65 y reported being able to drive at baseline	73,8	6,5	N.R.	48,3	1998	12	7	Volunteering {Yes vs. No}	Voluntary work carried out in the past 12 months	Formal	4788	34,6	N.R.
Curl et al. [25]	HRS^t^	USA	Respondents and spouses, aged ≥65 y, able to drive at baseline	73,9^u^/ 71,5^v^	5,4^u^ / 5,0^v^	N.R.	50,0	1998	12	7	Volunteering {Yes vs. No}	Voluntary work carried out in the past 12 months	Formal	2914^w^	40,0^u^ */* 41,5^v^	N.R.
Einolf & Philbrick [26]	PSID^x^	USA	Individuals never married at baseline	N.R.	N.R.	N.R.	N.R.	2003	2	2	-Volunteering {Yes vs. No} -Religious volunteering {Yes vs. No}	“How often did you volunteer at or through….”	Formal	452^y^; 610^z^	Rates at baseline and follow-up are not presented. Average rates for the two waves: 25,3%^aa^; 15,5%^ab^	
Hank & Erlinghagen [27]	SHARE^ac^	11 European countries	Individuals aged ≥50 y	N.R.	N.R.	N.R.	N.R.	2004/2005	2	2	- Volunteer engagement - Volunteer cessation	“Have you done any of these activities in the last month?” - “done voluntary or charity work”	Formal	18,057	10,0	10,8
Johnston [28]	ACL^ad^	USA	Individuals aged 25 and older living in the contiguous US.	54,0^ae^	N.R.	N.R.	54,0	1986	16	4	- Volunteering {Yes vs. No} - Religious institution volunteering {Yes vs. No} - Nonreligious institution volunteering {Yes vs. No}	Volunteer work done in the last year	Formal	1283^af^; 983^ag^; 1272^ah^	40,0	53,0
Lim & Mac Gregor [29]	FM^ai^	USA	Respondents who report that they do not attend religious services on a regular basis	47,3	16,0	N.R.	47,0	2006	5	2	Volunteering {Yes vs. No}	Volunteering in the past 12 months	Mixed	510	46,0	51,0
McNamara & Gonzales [30]	HRS^t^	USA	Individuals aged 50–80	63,0^aj^	N.R.	N.R.	58,7	2000/2001	8	5	-Volunteer engagement -Volunteer cessation	“Have you spend any time in the past 12 months doing volunteer work for charitable organizations?”	Formal	4611^ak^; 2961^al^	45,1	N.R.
Mike et al. [31]	HRS^t^	USA	Individuals ≥50 y, not volunteering and currently working/unemployed/retired	71,9	10,37	N.R.	54,0	2006/2008	2	2	Volunteering {Yes vs. No}	“Have you spent any time in the past year volunteering?”	Mixed	5017	0,0	13,6
Nesbit [32]	PSID^x^	USA	Household heads and their spouses	44,0	N.R.	N.R.	55,0	2003	2	2	-Religious volunteering {Yes vs. No} -Secular volunteering {Yes vs. No)	Volunteering in the last year	Formal	11299^am^; 11354^an^	27,0	29,0
Okun et al. [33]	ACL^ad^	USA	Individuals aged ≥65 y, reported volunteering in the past year	71,9	5,5	N.R.	71,0	1986	3	2	Volunteer cessation	Having done volunteer work in the last 12 months	Formal	380	100,0	61,0
Parkinson [34]	ALSWH^ao^	Australia	Women aged 70–75 y	N.R.	N.R.	N.R.	100	1996	9	4	Volunteering {Yes vs. No}	“Do you do any volunteer work for any community or social organizations?”	Mixed	7088	N.R.	24,5
Pavlova & Silbereisen [35]	Jena study^ap^	Germany	Individuals aged 16–43 and 56–75 years	38,1^aq^ / 60,2^ar^	3,9^aq^ / 3,9^ar^	N.R.	57,4^aq^ / 44,6^ar^	2005^ar^/2009^as^	1	2	-Volunteer engagement -Volunteer cessation	Participation in voluntary work in the past 12 months	Formal	1560^aq^; 518^ar^	20,6^ar^; 34.5^as^	31,3
Pavlova & Silbereisen [36]	Jena Study^ap^	Germany	Individuals aged 56–75 years	65,9	5,8	56–76	52,4	2009	1	2	Volunteering {Yes vs. No}	Participation in voluntary work in the past 12 months	Formal	602	32,5	35,9
Son & Wilson [37]	MIDUS^r^	USA	English speaking adults aged 25–74 y, living in the coterminous US	42,8	12,5	N.R.	55,0	1995	10	2	Volunteering {Yes vs. No}	“On average, about how many hours do you spend per month doing volunteer work?”	Formal	3257	39,0	43,0
Son & Wilson [38]	MIDUS^r^	USA	English speaking adults aged 25–74 y, living in the coterminous US	42,8	12,5	N.R.	55,0	1995	10	2	Volunteering {Yes vs. No}	“On average, about how many hours do you spend per month doing volunteer work?”	Formal	3257	39,0	43,0
Son & Wilson [39]	MIDUS^r^	USA	English speaking adults aged 25–74 y, living in the coterminous US	42,8	12,5	N.R.	55,0	1995	10	2	Volunteering {Yes vs. No}	“On average, about how many hours do you spend per month doing volunteer work?”	Formal	3257	39,0	43,0
Voorpostel & Coffé [40]	SHP^as^	Switzer-land	Adults aged 18–60 y	43,6^at^ /44,2^au^	12,0^at^ /11,8^au^	18–60	55,0	1999	8	9	Volunteering {Yes vs. No}	“Do you have honorary or voluntary activities within an association, an organization or an institution?”	Formal	8185^av^	42,5^aw^ / 31,6^ax^	39,5^aw^ / 29,5^ax^
Voorpostel & Coffé [41]	SHP^as^	Switzer-land	Adults aged 18–26 y, no change in partnership of parents during study	21,0	2,4	18–26	47,0	1999	10	11	Volunteering {Yes vs. No}	“Do you have honorary or voluntary activities within an association, an organization or an institution?”	Formal	3199^ay^	Volunteering rates at baseline and follow-up are not presented. The average overall volunteering rate for the two waves is 34,9	

^a^All included studies represent (subgroups of) the general population. Specification of subgroups is provided here

^b^Measured at baseline, unless denoted otherwise

^c^Represents the measurement in the year that is used as baseline for the analysis

^d^Type: Formal volunteering (through an organization), Mixed (no distinction between formal and informal volunteering, or type of volunteering (formal/informal) not specified

^e^Social Relations and Health over the Life Course

^f^United States of America

^g^Not Reported

^h^British Household Panel Survey

^i^United Kingdom

^j^Giving in the Netherlands Panel Study

^k^The Netherlands

^l^Volunteers

^m^Non-volunteers

^n^Longitudinal Aging Study Amsterdam

^o^Cohort 1

^p^Cohort 2

^q^For each cohort

^r^Survey of Midlife Development in the United States

^s^Not applicable

^t^Health and Retirement Study

^u^Husbands

^v^Wives

^w^1457 couples

^x^Panel Study of Income Dynamics

^y^Males

^z^Females

^aa^Volunteering

^ab^Religious volunteering

^ac^Survey of Health, Ageing and Retirement in Europe

^ad^American’s Changing Lives Study

^ae^Approximately

^af^Volunteering sample

^ag^Religious institution volunteering sample

^ah^Nonreligious institution volunteering sample

^ai^Faith Matters Survey

^aj^Mean age is measured over all included waves

^ak^Outcome engagement

^al^Outcome cessation

^am^Religious volunteering

^an^Secular volunteering

^ao^Australian Longitudinal Study On Womens Health

^ap^Jena Study on Social Change and Human Development

^aq^Sample 1 Age group 30–43

^ar^Sample 2 Age group 56–75

^as^Switzerland Household Panel

^at^Males, measured at follow-up

^au^Females, measured at follow-up

^av^3692 males and 4493 females

^aw^Males

^ax^Females

^ay^1788 respondents and their mothers and 1331 respondents and their fathers

Several articles were based on the same study samples. Four articles were based on data from the Survey of Midlife Development in the United States [22, 37–39]. Another four articles were based on data of the Health and Retirement Study [24, 25, 30, 31]. Two articles used data from the Jena Study on Social Change and Human Development [35, 36]. Moreover, two articles used data from the Switzerland Household Panel [40, 41]. Finally, two articles were based on the American Changing Lives survey [28, 33].

### Likelihood of risk of bias

The results of the risk of bias assessment of included studies are presented in Table 2.

**Table 2:** Risk of Bias table (Based on QUIPS^a^)

Author	1. Study participation		2. Study attrition		3. Determinant measurement			4. Outcome measurement		5. Study confounding										6. Statistical analysis and reporting		
										5a. Confounders measured						5d. Confounders accounted for in analysis						
	1a. Consecutive series of participants	1b. Adequate participation rate (> 70%)	2a. Adequate follow-up rate (≥80%)	2b. No important differences between participants and drop-out	3a. ≥70% complete data for each determinant	3b. Method and setting of the measurement is the same for all study participants	3c. Appropriate methods of imputation	4a. Outcome measurement truly captures volunteering	4b. Method and setting of measurement is the same for all study participants	5a1. Age	5a2. Socioeconomic Status	5a3. Gender	5a4. Participation in voluntary work at baseline	5b. Method and setting of measurement is the same for all study participants	5c. Appropriate methods of imputation	5d1. Age	5d2. Socioeconomic Status	5d3. Gender	5d4. Participation in voluntary work at baseline	6a. Statistical model adequate for study design	6b. No overfitting	6c. No selective reporting of results
Ajrouch et al. [18]	+	+	–	?	+	+	N.A.^b^	+	+	+	+	+	–	+	N.A.	+	+	+	–	+	+	+
Bartels et al. [19]	+	?	?	?	?	+	?	+	+	–	+	+	+	+	?	–	+	+	+	+	+	+
Bekkers [20]	–	?	–	+	?	+	?	+	+	+	+	+	+	+	?	–	–	–	–	+	+	+
Broese van Groenou & Van Tilburg [21]	+	–	–	?	?	+	?	+	+	+	+	+	+	+	?	+	+	+	+	+	+	+
Choi & Chou [22]	+	–	–	+	?	+	?	+	+	+	+	+	+	+	?	+	+	+	+	+	+/−^c^	+
Cramm & Nieboer [23]	–	–	–	–	?	+	N.A.	+	+	+	+	+	+	+	N.A.	+	+	+	+	+	–	+
Curl et al. [24]	+	?	?	?	+	+	N.A.	+	+	+	+	+	+	+	N.A.	+	+	+	+	+	+	+
Curl et al. [25]	+	?	?	?	+	+	N.A.	+	+	+	+	+	+	+	N.A.	+	+	+	+	+	+	+
Einolf & Philbrick [26]	+	?	+	?	?	+	?	+	+	+	+	+	+	+	?	+	+	+	+	+	+	+
Hank & Erlinghagen [27]	+	–	–	?	?	?	?	+	?	+	+	+	+	?	?	+	+	+	+	+	+	+
Johnston [28]	+	–	–	?	?	+	?	+	+	+	+	+	+	+	?	–	+	+	+	+	+	+
Lim & Mac Gregor [29]	+	?	–	?	?	+	?	+	+	+	+	+	+	+	?	+	+	+	+	+	+	+
McNamara & Gonzales [30]	+	?	?	?	+	+	+	+	+	+	+	+	+	+	+	+	+	+	+	+	+	+
Mike et al. [31]	+	?	?	?	+	+	N.A.	+	+	+	+	+	+	+	N.A.	+	+	+	+	+	+	+
Nesbit [32]	+	?	?	?	?	+	?	+	+	+	+	+	+	+	?	+	+	+	+	+	+	+
Okun et al. [33]	+	?	?	?	+	+	N.A.	+	+	+	+	+	+	+	N.A.	+	+	+	+	+	+	+
Parkinson [34]	+	?	–	?	?	+	?	+	+	+	+	+	+	+	?	+	+	+	+	+	+	+
Pavlova & Silbereisen [35]	+	+/−^d^	?^e^	−/+^f^	+	+	+	+	+	+	+	+	+	+	+	+	+	+	+	+	+	+
Pavlova & Silbereisen [36]	+	–	–	+	+	+	+	+	+	+	+	+	+	+	+	+	+	+	+	+	+	+
Son & Wilson [37]	+	+	–	?	?	+	+	+	+	+	+	+	+	+	+	+	+	+	+	+	+	+
Son & Wilson [38]	+	+	–	?	?	+	+	+	+	+	+	+	+	+	+	+	+	+	+	+	+	+
Son & Wilson [39]	+	+	–	?	?	+	+	+	+	+	+	+	+	+	+	+	+	+	+	+	+	+
Voorpostel & Coffé [40]	+	?	–	?	?	+	?	+	+	+	+	+	+	+	?	+	+	+	+	+	+	+
Voorpostel & Coffé [41]	+	?	–	?	?	+	?	+	+	+	+	+	+	+	?	+	+	+	+	+	+	+

^a^*QUIPS* Quality of Prognosis Studies in Systematic Reviews. Assessment: + (Yes) (represents low risk of bias); - (No) (represents high risk of bias); ? (Unclear) (represents uncertain risk of bias, insufficient information was available to assess the risk of bias)

^b^Not Applicable

^c^For the outcome volunteer engagement (starting) there is no over fitting, so low risk of bias, but for the outcome volunteer cessation (quitting), there is slight over fitting of the model, so high risk of bias

^d^Baseline participation in the first sample (age group 16-43) was adequate (77%), but the baseline participation in the second sample (age group 56-75) not (52,9%)

^e^No information is provided on the follow-up rates. However, the second sample (age group 56-75) is the same as the sample used in Pavlova et al. 2016 and attrition is higher than 20%

^f^Attrition in the first sample (age group 16-43) was selective w.r.t. volunteering at T1, for the second sample (age group 56-75) attrition was not selective w.r.t. volunteering at T1

The risk of bias varied substantially. Most methodological flaws (i.e. high risk of bias) were found for (2a) adequate follow-up rate (62.5% high risk of bias), and (1b) adequate participation rate (29.2% high risk of bias). The inter-rater agreement was good (agreement 91.7% (484/528); kappa statistic: 0.78) [14].

### Determinants of participation in voluntary work

Meta-analyses were conducted for a total of 20 determinants (see Appendix 4). For each determinant, all studies reporting an association between the determinant and the outcome are listed in the appendix, as well as the studies selected for inclusion in the meta-analysis.

#### Demographic factors

**Fig. 2:**
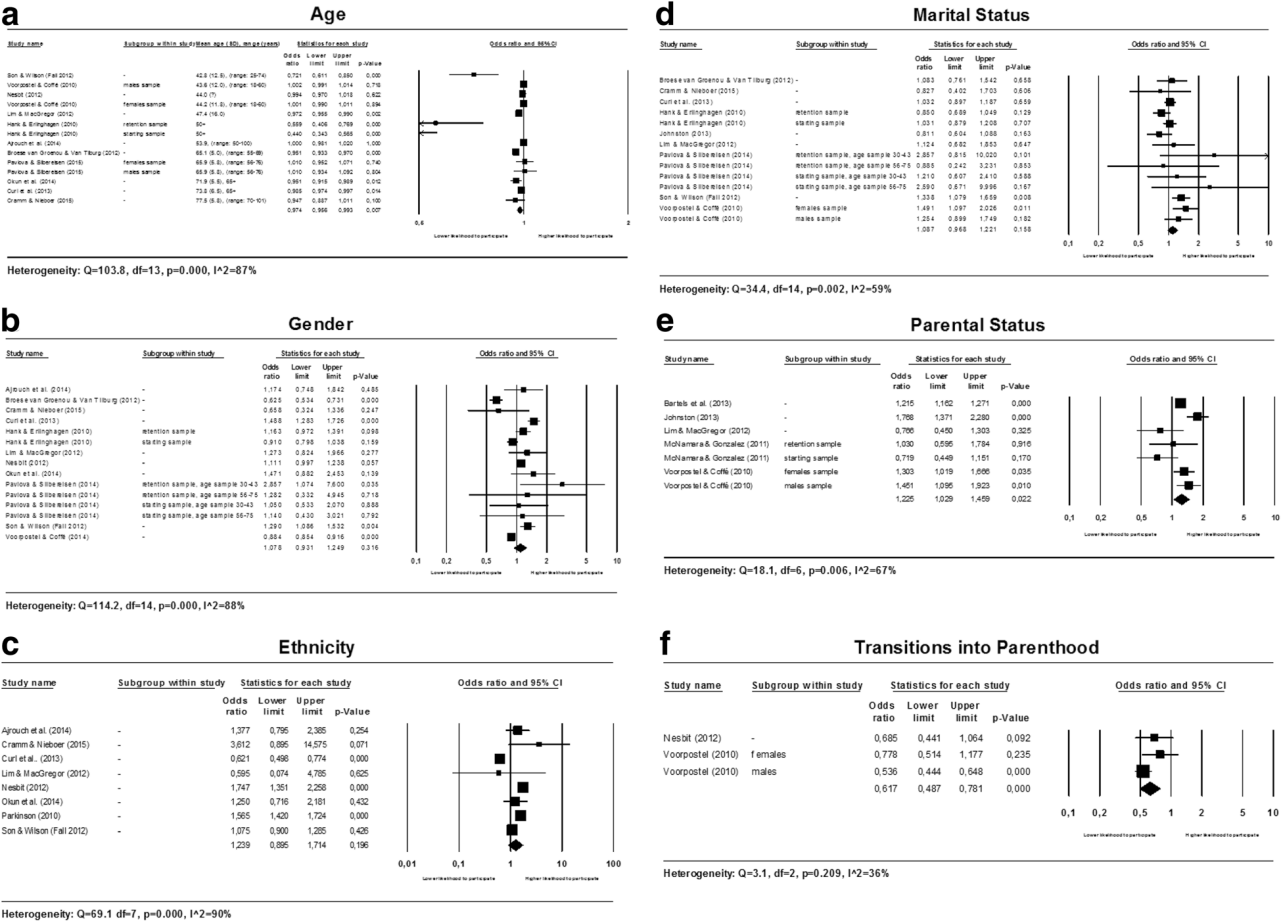
Forest plots for demographic factors

The following demographic factors are studied in relationship to participation in voluntary work: age, gender, ethnicity, marital status and parental status. Forest plots for all demographic factors are presented in multi panel Fig. 2 below.

##### Age

The mean age at baseline of the studies included in the meta-analysis varied from 42.8 years (range 25 to 74 years) [39] to 77.5 years (range 70 to 101 years) [23]. The results of the meta-analysis are heterogeneous (see Fig. 2a). Six studies [11, 21, 24, 26, 30, 36] (out of eleven) found that older people are less likely to volunteer, no associations in the opposite direction were found. Sources of heterogeneity were explored by conducting meta-regression analyses and subgroup analyses (see Table 3).

**Table 3:** Univariate random effects meta-regression (methods of moments) and subgroup analyses for AGE

			Subgroup analyses				Results from meta-regression		
			Results		Heterogeneity				
Variable	Subgroup	Number of studies	OR	95% CI	*P*-value	*I* ^2^	Coefficient	SD	*P*-value
Outcome measurement	Mixed	2	0.986	0.959–1.013	0.037	77%	Reference		
	Formal	9 (12 different samples)	0.969	0.946–0.992	0.000	89%	−0.0168	0.0251	0.504
Determinant measurement	Dichotomous	1 (2 different samples)	0.485	0.385–0.611	0.248	25%	Reference		
	Continuous	10 (12 different samples)	0.983	0.969–0.996	0.000	78%	0.7122	0.1014	0.000
Proportion of volunteers (%) in baseline study sample	*Continuous*	10^a^ (13 different samples)	0.970	0.950–0.991	0.000	88%	0.0000	0.0001	0.591
	0–100%	9 (11 different samples)	0.985	0.971–0.999	0.000	79%	Reference		
	0%	1	0.440	0.343–0.565	N.A.	N.A.	−0.8053	0.1292	0.000
	100%	2	0.747	0.444–1.256	0.001	91%	−0.0500	0.0292	0.087
Mean age at baseline	*Continuous*	10^b^ (12 different samples)	0.983	0.969–0.996	0.000	78%	−0.0000	0.0000	0.200
	≤ 55 years	5 (6 different samples)	0.991	0.975–1.007	0.000	79%	Reference		
	> 55 years	6 (8 different samples)	0.944	0.904–0.986	0.000	89%	−0.0296	0.0198	0.135
Continent	USA	6	0.978	0.959–0.998	0.001	77%	Reference		
	Europe	5 (8 different samples)	0.966	0.933–1.000	0.000	91%	−0.0026	0.0210	0.900
Year of baseline measurement	*Continuous*	11 (14 different samples)	0.989	0.984–0.995	0.000	87%	0.0006	0.0014	0.686
	< 2006	8 (10 different samples)	0.970	0.948–0.993	0.000	91%	Reference		
	≥ 2006	3 (4 different samples)	0.975	0.959–0.991	0.388	1%	0.0110	0.0230	0.631
Risk of bias items									
Study participation	Unclear/high risk of bias	9 (12 different samples)	0.975	0.956–0.995	0.000	88%	Reference		
	Low risk of bias	2	0.858	0.623–1.192	0.000	93%	−0.0099	0.0315	0.754
Study confounding	Unclear/high risk of bias	1	1.000	0.981–1.020	N.A.	N.A.	Reference		
	Low risk of bias	10 (13 different samples)	0.970	0.950–0.991	0.000	88%	−0.0303	0.0331	0.360

^a^The study of Ajrouch et al. (2014) is not included in this analysis, because the proportion of volunteers (%) in the baseline study sample is not reported

^b^The study of Hank & Erlinghagen (2010) is not included in this analysis, because the mean age at baseline is not reported

The meta-regression shows that differences in the proportion of volunteers in the baseline sample affect the association between age and participation in voluntary work. The negative coefficient from the meta-regression (− 0.8053, *p* = 0.000) shows that the inverse association between age and the likelihood to volunteer is stronger in the non-volunteer sample than in the subgroup of samples in which the proportion of volunteers lies between 0 and 100%. Therefore, the results indicate that the likelihood to participate in voluntary work declines with age, and that especially the likelihood to take-up voluntary work (for individuals not volunteering at baseline) strongly decreases with age.

##### Gender (female)

Two studies (out of eleven) included in the meta-analysis did not report the percentage of females in the baseline study sample [21, 27]. The percentage of females in the baseline study samples of the other included studies ranged from 44.6% [32] to 71.0% [33] (heterogeneous results; see Fig. 2b).

The results of the meta-regression (Table 4) showed that differences in the continent (Europe versus USA) of the study sample explain heterogeneity in the association between gender and participation in voluntary work.

**Table 4:** Univariate random effects meta-regression (methods of moments) and subgroup analyses for GENDER (female)

			Subgroup analyses				Results from meta-regression		
			Results		Heterogeneity				
Variable	Subgroup	Number of studies	OR	95% CI	*P*-value	*I* ^2^	Coefficient	SD	*P*-value
Outcome measurement	Mixed	2	1.224	0.895–1.674	0.800	0%	Reference		
	Formal	9 (13 different samples)	1.061	0.907–1.243	0.000	89%	−0.1424	0.2379	0.550
Proportion of volunteers (%) in baseline study sample	*Continuous*	9^a^ (13 different samples)	1.099	0.917–1.317	0.000	89%	0.0004	0.0003	0.177
	0–100%	8	1.038	0.805–1.268	0.000	93%	Reference		
	0%	2 (3 different samples)	0.918	0.808–1.043	0.836	0%	−0.0689	0.2266	0.761
	100%	3 (4 different samples)	1.306	1.000–1.705	0.296	19%	0.2926	0.2156	0.175
Mean age at baseline	*Continuous*	10^b^ (13 different samples)	1.109	0.920–1.337	0.000	86%	−0.0000	0.0006	0.952
	≤ 55 years	6 (8 different samples)	1.136	0.939–1.374	0.000	85%	Reference		
	> 55 years	6 (7 different samples)	1.023	0.765–1.367	0.000	90%	−0.1296	0.1695	0.445
Continent	USA	6	1.279	1.120–1.460	0.063	52%	Reference		
	Europe	5 (9 different samples)	0.906	0.770–1.067	0.000	77%	−0.3531	0.1135	0.002
Year of baseline measurement	*Continuous*	11^c^ (15 different samples)	1.078	0.931–1.249	0.000	88%	0.0008	0.0126	0.951
	< 2006	9 (11 different samples)	1.083	0.924–1.270	0.000	91%	Reference		
	≥ 2006	3 (4 different samples)	1.084	0.775–1.516	0.474	0%	−0.0301	0.2334	0.897
Risk of bias items									
Study participation	Unclear/high risk of bias	9 (11 different samples)	1.025	0.871–1.205	0.000	89%	Reference		
	Low risk of bias	3 (4 different samples)	1.288	1.094–1.515	0.383	2%	0.2436	0.1809	0.178
Study confounding	Unclear/high risk of bias	1	1.174	0.748–1.842	N.A.	N.A.	Reference		
	Low risk of bias	10 (14 different samples)	1.073	0.922–1.250	0.000	89%	−0.0898	0.3302	0.786

^a^The studies of Ajrouch et al. (2014) and Voorpostel & Coffé (2014) are not included in this analysis, because the proportion of volunteers (%) in the baseline study sample is not reported

^b^The study of Hank & Erlinghagen (2010) is not included in this analysis, because the mean age at baseline is not reported

^c^The study of Broese van Groenou & Van Tilburg (2012) includes two different samples in the analyses. For one of the samples, the year of baseline measurement is 1992, for the other sample, the year of baseline measurement is 2002. No separate results for the two samples are provided. In this specific analysis, we took 1992 as the year of baseline measurement, although this actually only is the case for the first sample

The negative coefficient (− 0.3531; *p* = 0.002) from the meta-regression for Europe (USA as reference group) shows that the likelihood of females (as opposed to males) to participate in voluntary work is higher in the USA than in Europe. In the studies conducted in the USA [18, 24, 29, 32, 33, 39], a positive association between being female and participation in voluntary work was found (OR: 1.279; 95% CI: 1.120–1.460; results are heterogeneous (*I*^2^= 52%)). In the studies conducted in Europe [21, 23, 27, 35, 41], no association between gender and participation in voluntary work was found (OR: 0.906; 95% CI: 0.770–1.067; results are heterogeneous (*I*^2^= 77%)). Having a closer look at the subgroups of studies conducted in the USA and in Europe shows that (a) in Europe no consistent association between gender and participation in voluntary work was found (both positive as well as negative associations between gender and participation in voluntary work were found) whereas (b) in the subgroup of studies conducted in the USA, all odds ratios for the association between being female and participation in voluntary work are greater than one, indicating a greater likelihood of females (as opposed to males) to participate in voluntary work.

##### Ethnicity (white)

The results of the studies investigating the association between ethnicity and participation in voluntary work are heterogeneous and inconsistent (see Fig. 2c).

Heterogeneity could be explained by conducting subgroup analyses for differences in (a) year of baseline measurement (no association for the studies with a baseline measurement after 2005 [23, 29] (OR: 1.743; 95% CI: 0.308–9.877) and (b) the risk of bias for the domain study participation (no association for the studies with low risk of bias [18, 39] (OR: 1.101; 95% CI: 0.929–1.034). Forest plots are available upon request.

##### Marital status (married/partnered)

The results of the meta-analysis for marital status are heterogeneous and inconsistent (see Fig. 2d).

Sources of heterogeneity were explored by conducting meta-regression and subgroup analyses (see Table 5).

**Table 5:** Univariate random effects meta-regression (methods of moments) and subgroup analyses for MARITAL STATUS (married/partnered)

			Subgroup analyses				Results from meta-regression		
			Results		Heterogeneity				
Variable	Subgroup	Number of studies	OR	95% CI	*P*-value	*I* ^2^	Coefficient	SD	*P*-value
Outcome measurement	Mixed	1	1.124	0.682–1.853	N.A.	N.A.	Reference		
	Formal	9 (14 different samples)	1.053	0.931–1.192	0.001	62%	−0.0650	0.3067	0.832
Proportion of volunteers (%) in baseline study sample	*Continuous*	9^a^ (14 different samples)	1.087	0.968–1.221	0.045	43%	− 0.0002	0.0002	0.385
	0–100%	8 (9 different samples)	1.071	0.917–1.250	0.001	70%	Reference		
	0%	2 (3 different samples)	1.052	0.902–1.227	0.381	0%	0.0468	0.1907	0.806
	100%	2 (3 different samples)	1.080	0.564–2.066	0.175	43%	−0.1253	0.2095	0.550
Mean age at baseline	*Continuous*	8^b^ (12 different samples)	1.147	1.001–1.315	0.112	35%	− 0.0008	0.0004	0.030
	≤ 55 years	6 (8 different samples)	1.140	0.911–1.427	0.000	76%	Reference		
	> 55 years	5 (7 different samples)	0.999	0.913–1.092	0.539	0%	−0.1477	0.1419	0.300
Continent	USA	4	1.065	0.870–1.304	0.049	62%	Reference		
	Europe	6 (11 different samples)	1.054	0.904–1.230	0.009	57%	−0.0106	0.1314	0.936
Year of baseline measurement	*Continuous*	10 (15 different samples)	1.055	0.937–1.188	0.002	59%	0.0088	0.0096	0.361
	< 2006	8 (11 different samples)	1.055	0.928–1.199	0.000	69%	Reference		
	≥ 2006	3 (4 different samples)	1.081	0.742–1.575	0.522	0%	0.0295	0.2251	0.896
Duration of follow-up	*Continuous*	10 (15 different samples)	1.055	0.937–1.188	0.002	59%	−0.0111	0.0115	0.335
	≤ 3 years	3 (7 different samples)	0.990	0.830–1.180	0.274	20%	Reference		
	4–7 years	2	1.096	0.822–1.463	0.905	0%	0.0776	0.2315	0.737
	≥ 8 years	5 (6 different samples)	1.082	0.896–1.306	0.000	81%	0.0580	0.1499	0.699
Risk of bias items									
Study participation	Unclear/high risk of bias	9 (12 different samples)	1.004	0.897–1.124	0.019	52%	Reference		
	Low risk of bias	2 (3 different samples)	1.353	1.105–1.657	0.478	0%	0.3106	0.1563	0.047
Study confounding	Unclear/high risk of bias	2	0.846	0.766–0.935	0.763	0%	Reference		
	Low risk of bias	8 (13 different samples)	1.115	0.994–1.252	0.083	38%	0.2803	0.1113	0.012

^a^The study of Bartels et al. (2013) is not included in this analysis, because the proportion of volunteers (%) in the baseline study sample is not reported

^b^The studies of Bartels et al. (2013) and Hank & Erlinghagen (2010) are not included in this analysis, because the mean age at baseline is not reported

The results of the meta-regression show that differences in (a) mean age at baseline and (b) the risk of bias for the domains study participation and study confounding affect the association between marital status and participation in voluntary work.

Firstly, the pooled estimate of the subgroup of the eight studies [21, 23, 24, 28, 29, 35, 39, 40] for which information on the mean age at baseline is available, shows that married people are more likely to participate in voluntary work than unmarried people (OR: 1.147; 95% CI: 1.001–1.315; results are homogenous (*I*^2^= 35%)). The negative coefficient (− 0.0008; *p* = 0.030) from the meta-regression shows that the positive association between being married and participation in voluntary work declines with age; i.e. being married as a determinant of participation in voluntary work declines in importance with age.

Secondly, the positive coefficients from the meta-regression for the risk of bias domains study participation (0.3106; *p* = 0.047) and study confounding (0.2803; *p* = 0.012) show that the association between being married and participation in voluntary work is stronger in studies with low risk of bias on these domains than for the studies with unclear/high risk of bias.

Although we did not find an overall association between marital status and participation in voluntary work, several subgroups of studies point towards a positive association between being married/partnered and the likelihood to volunteer. The meta-regression shows that as age increases, the association between being married/partnered and the likelihood to participate in voluntary work gets less strong. Our findings are in line with earlier research, showing that being married is positively associated to participation in voluntary work; but associations between marital status and volunteering after retirement are inconsistent [9].

##### Parental status

The results of the studies investigating the association between parental status and participation in voluntary work are heterogeneous (see Fig. 2e). Heterogeneity could not be explained by conducting subgroup analyses. Three studies [19, 28, 40] (out of five) found a positive association between having children and participation in voluntary work and no negative associations were found. Although no firm conclusion can be drawn from these results, the results seem to indicate that parents with children in their household are more likely to volunteer.

Two articles [32, 40] reported estimates for the association between a transition into parenthood and participation in voluntary work. The pooled estimate of these two studies shows that individuals who recently had a child were less likely to participate in voluntary work than individuals who did not experience the birth of a child in the household recently (OR: 0.617; 95% CI: 0.487 to 0.781) (see Fig. 2f).

#### Socioeconomic status

**Fig. 3:**
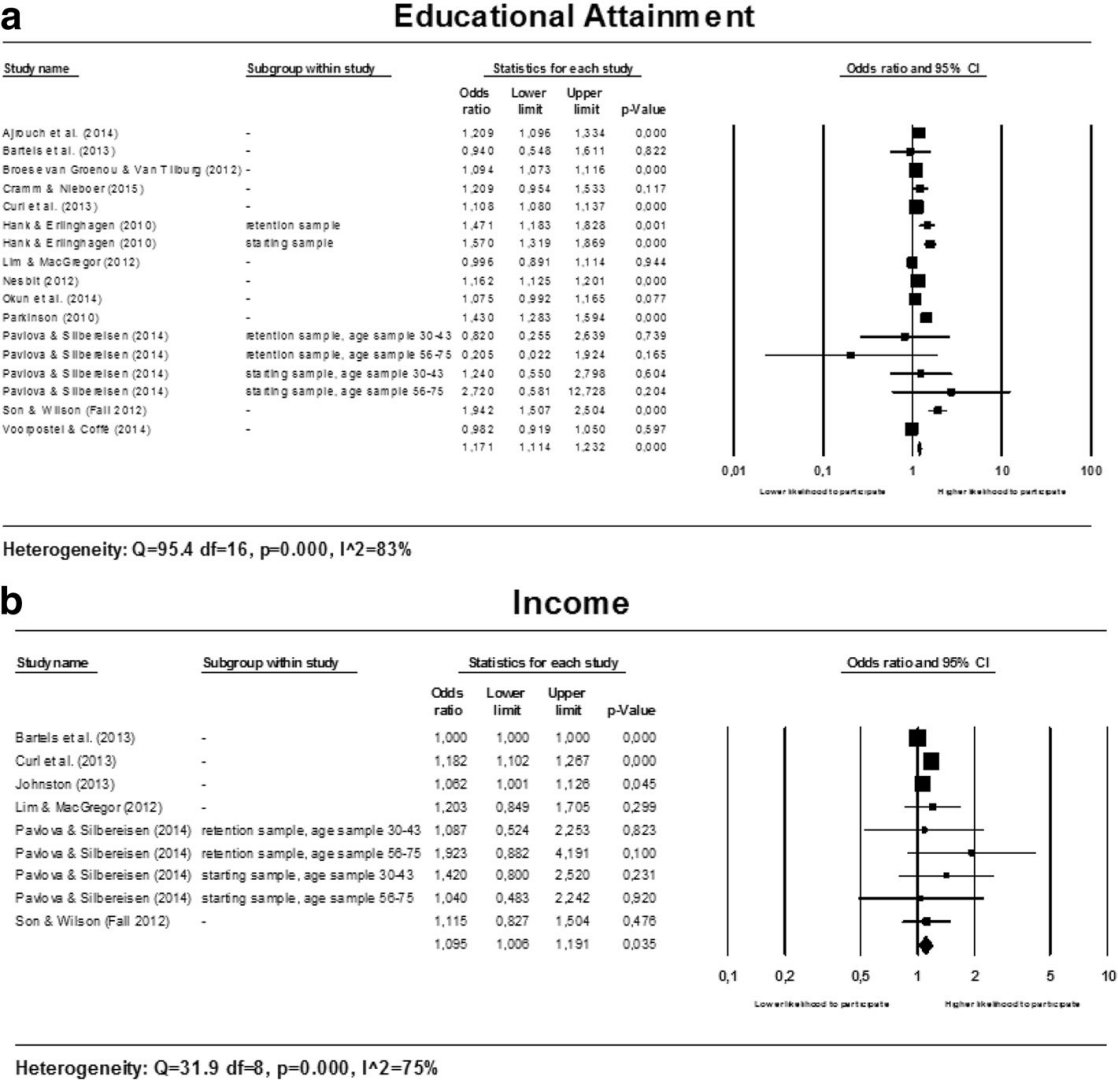
Forest plots for socioeconomic factors

Two factors related to socioeconomic status are studied in relationship to participation in voluntary work. Meta-analyses were conducted for educational attainment as well as income. The forest plots are presented in multi panel Fig. 3 below.

##### Educational attainment

The results of the meta-analysis for educational attainment are heterogeneous (see Fig. 3a). Seven studies [18, 21, 24, 27, 32, 34, 39] (out of thirteen) found that higher educated individuals are more likely to participate in voluntary work, no associations in the opposite direction were found. Sources of heterogeneity were explored by conducting meta-regression and subgroup analyses (see Table 6).

**Table 6:** Univariate random effects meta-regression (methods of moments) and subgroup analyses for EDUCATIONAL ATTAINMENT

			Subgroup analyses				Results from meta-regression		
			Results		Heterogeneity				
Variable	Subgroup	Number of studies	OR	95% CI	*P*-value	*I* ^2^	Coefficient	SD	*P*-value
Outcome measurement	Mixed	3	1.199	0.985–1.460	0.000	90%	Reference		
	Formal	10 (14 different samples)	1.153	1.094–1.215	0.000	81%	−0.0335	0.0591	0.571
Determinant measurement	Dichotomous	5 (9 different samples)	1.256	1.001–1.577	0.000	86%	Reference		
	Continuous	8	1.130	1.082–1.179	0.000	80%	−0.0922	0.0579	0.111
Proportion of volunteers (%) in baseline study sample	*Continuous*	9^a^ (13 different samples)	1.162	1.104–1.223	0.000	79%	−0.0001	0.0001	0.176
	0–100%	10	1.147	1.088–1.208	0.000	87%	Reference		
	0%	2 (3 different samples)	1.564	1.321–1.853	0.667	0%	0.3080	0.1100	0.005
	100%	3 (4 different samples)	1.171	0.870–1.577	0.024	68%	0.0083	0.0720	0.908
Mean age at baseline	*Continuous*	10^b^ (13 different samples)	1.111	1.064–1.161	0.000	77%	0.0001	0.0001	0.493
	≤ 55 years	7 (8 different samples)	1.148	1.025–1.286	0.000	84%	Reference		
	> 55 years	7 (9 different samples)	1.203	1.128–1.284	0.000	84%	0.0711	0.0576	0.217
Continent	USA	6	1.144	1.075–1.218	0.000	84%	Reference		
	Europe	6 (10 different samples)	1.186	1.055–1.333	0.000	77%	0.0110	0.0598	0.854
	Australia	1	1.430	1.283–1.594	N.A.	N.A.	0.2164	0.1049	0.039
Year of baseline measurement	*Continuous*	13 (17 different samples)	1.171	1.114–1.232	0.000	83%	N.A.		
	< 2006	11 (13 different samples)	1.187	1.125–1.252	0.000	86%	Reference		
	≥ 2006	3 (4 different samples)	1.081	0.852–1.372	0.132	47%	−0.1167	0.0810	0.150
Duration of follow-up	*Continuous*	13 (17 different samples)	1.171	1.114–1.232	0.000	83%	N.A.		
	≤ 3 years	5 (9 different samples)	1.241	1.114–1.382	0.003	66%	Reference		
	4–7 years	2	1.062	0.974–1.157	0.104	62%	−0.1728	0.1068	0.106
	≥ 8 years	6	1.225	1.081–1.389	0.000	91%	−0.0225	0.0840	0.789
Risk of bias items									
Study participation	Unclear/high risk of bias	3 (4 different samples)	1.144	1.089–1.203	0.000	84%	Reference		
	Low risk of bias	11 (13 different samples)	1.396	0.973–2.004	0.007	75%	0.1620	0.0766	0.034
Study confounding	Unclear/high risk of bias	2	1.199	1.089–1.321	0.387	0%	Reference		
	Low risk of bias	11 (15 different samples)	1.171	1.110–1.235	0.000	85%	−0.0105	0.0880	0.905

^a^The studies of Ajrouch et al. (2014), Bartels et al. (2013), Parkinson (2010) and Voorpostel & Coffé (2014) are not included in this analysis, because the proportion of volunteers (%) in the baseline study sample is not reported

^b^The studies of Bartels et al. (2013), Hank & Erlinghagen (2010) and Parkinson (2010) are not included in this analysis, because the mean age at baseline is not reported

Results show that the association between educational attainment and the likelihood to volunteer is stronger in (a) samples consisting of non-volunteers (compared to samples consisting of both volunteers and non-volunteers) (0.3080; *p* = 0.005), (b) the study conducted in Australia (compared to studies from the USA) (0.2164; *p* = 0.039) and (c) studies with low risk of bias on the domain study participation (compared to studies with high/unclear risk of bias) (0.1620; *p* = 0.034).

Although the results for the subgroups were heterogeneous, the pooled estimate of most studies point towards a positive association between educational attainment and participation in voluntary work. No contradictory results are found. Therefore, the results indicate that it is likely that there is a positive association between educational attainment and the likelihood to volunteer and this positive association seems to be especially strong for volunteer take-up.

##### Income

The meta-analysis for income gives heterogeneous results (see Fig. 3b). Two studies [24, 28] (out of six) found a positive association between income and participation in voluntary work, no associations in the opposite direction were found.

Subgroup analyses show that people with a higher income are more likely to participate in voluntary work if they are 55 years or older at baseline ((OR: 1.185; 95% CI: 1.106 to 1.270) [24, 35] or if they are living in the USA (OR: 1.121, 95% CI: 1.037 to 1.211) [24, 28, 29, 39]. For the studies with a low risk of bias on the domain study confounding (OR: 1.184; 95% CI: 1.109 to 1.265) [24, 29, 35, 39] this positive association between income level and the likelihood to participate in voluntary work was confirmed. No association between income and participation in voluntary work was found in the other subgroups with homogeneous results. Forest plots are available upon request.

#### Participation in productive activities

**Fig. 4:**
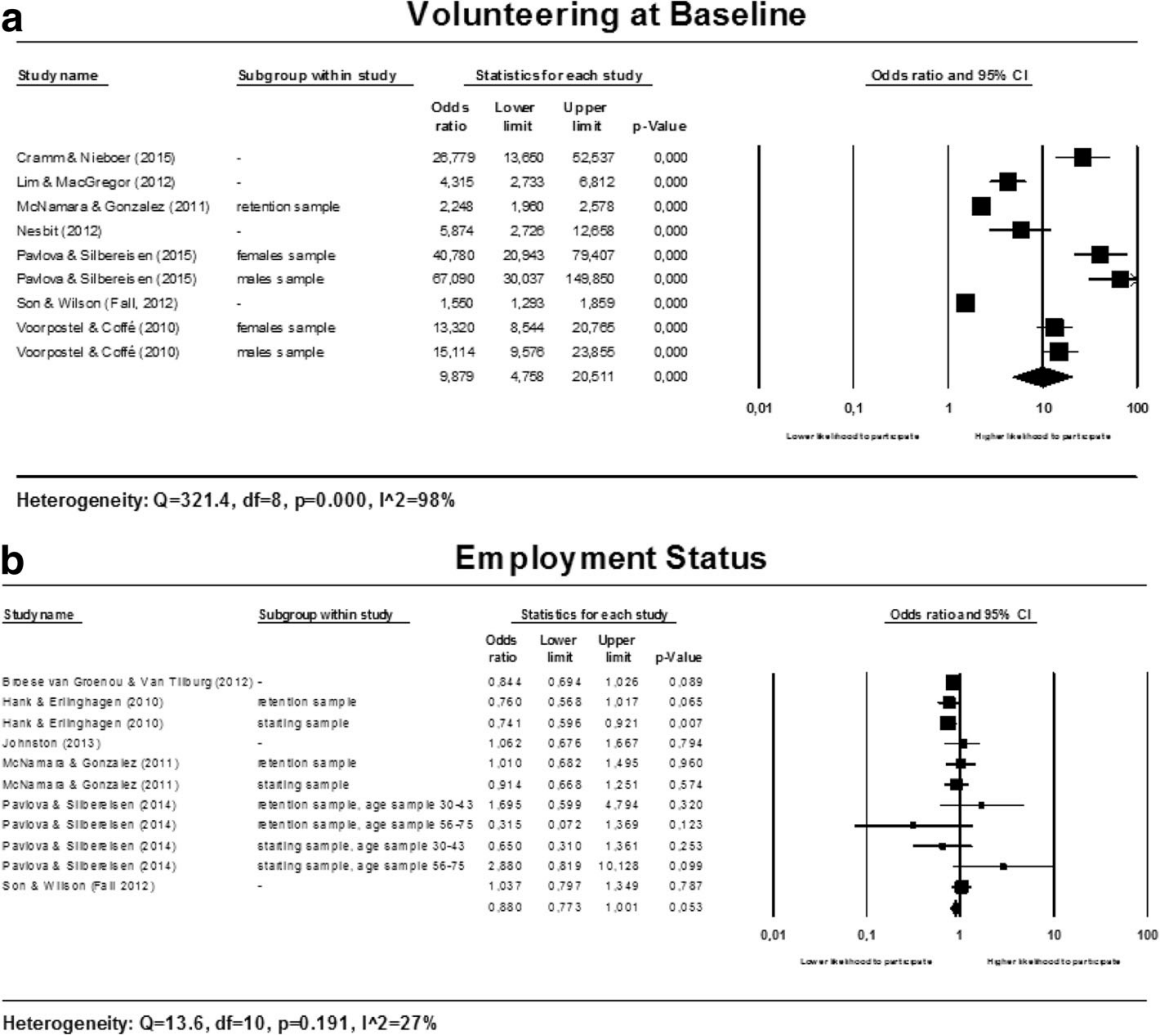
Forest plots for participation in productive activities

Two factors related to participation in productive activities are studied in relationship to participation in voluntary work. Meta-analyses were conducted for participation in voluntary work at baseline and for employment status. The forest plots are presented in multi panel Fig. 4 below.

##### Volunteering at baseline

Results for the meta-analysis are heterogeneous (see Fig. 4a) and heterogeneity could not be explained by subgroup analyses. However, the estimates of the included studies clearly show that volunteering at baseline is positively associated to participation at follow-up; all included studies found a positive association between volunteering at baseline and volunteering at follow-up. No firm conclusion can be drawn about the magnitude of the effect.

##### Employment status

Results from the meta-analysis for employment status are homogeneous (see Fig. 4b). The pooled estimate shows no association between employment status and participation in voluntary work (OR: 0.880; 95% CI: 0.773 to 1.001); however, the *p*-value of 0.053 shows that the association is boundary significant.

#### Health status

**Fig. 5:**
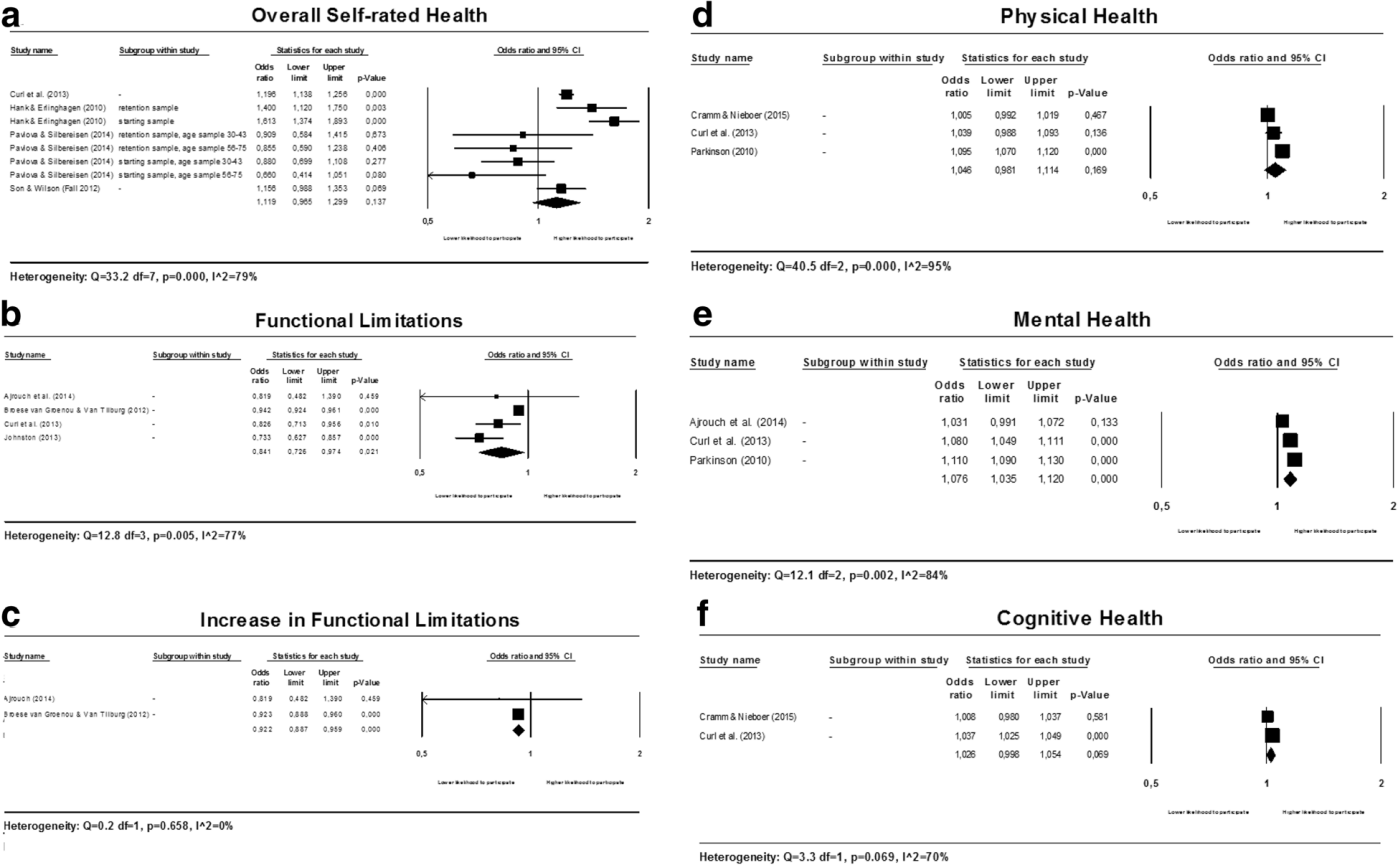
Forest plots for health status

Five factors related to individual health status are studied in relationship to participation in voluntary work. Separate meta-analyses were conducted for overall self-rated health, (increase in) functional limitations, physical health, mental health and cognitive health. Forest plots for all factors related to individual health status are presented in multi panel Fig. 5 below.

##### Overall self-rated health

The meta-analysis for overall self-rated health shows that results are heterogeneous (see Fig. 5a).

Heterogeneity between the results of the included studies could be explained by differences in (a) participation in voluntary work (%) at baseline, (b) continent of the study sample and (c) duration of follow-up. The pooled estimate of the two studies [24, 39] with a baseline participation rate between 0% and 100%, a long duration of follow-up (≥ 8 years) and that are conducted in the USA shows that people with a better overall self-rated health are more likely to participate in voluntary work (OR: 1.192; 95% CI: 1.137 to 1.249). Forest plots are available on request.

##### Functional limitations

Three large studies [21, 24, 28] found a strong negative association between functional limitations and the likelihood to participate in voluntary work, one small study [18] did not find an association. Although the results are heterogeneous, the results clearly indicate that the degree of functional limitations is inversely associated with participation in voluntary work (see Fig. 5b).

The pooled estimates of the two studies [18, 28] for which the mean age at baseline was 55 years or below (OR: 0.740, 95% CI: 0.636 to 0.860), the three studies [18, 24, 28] conducted in the USA (OR: 0.782; 95% CI: 0.705 to 0.869), and the two studies [24, 28] with a long duration of follow-up (≥ 8 years) (OR: 0.781; 95% CI: 0.695 to 0.877) consistently show that individuals with more functional limitations are less likely to participate in voluntary work. Forest plots are available on request.

Two studies [18, 21] reported an estimate for the association between an increase in the degree of limitations in functional health and participation in voluntary work (see Fig. 5c). The pooled estimate of these two studies shows that increases in functional limitations are associated with a lower likelihood to participate in voluntary work (OR: 0.922; 95% CI: 0.887 to 0.959).

##### Physical health

The results for the association between physical health and participation in voluntary work are heterogeneous (see Fig. 5d).

Pooling the estimates of the studies with formal volunteering as the outcome (as opposed to the mixed type of volunteering) and the estimates of the studies with low risk of bias on the domain study confounding leaves us with the same subgroup of two studies [23, 24]. No association between physical health and participation in voluntary work was found (OR: 1.013; 95% CI: 0.985 to 1.041) (forest plot is available on request).

##### Mental health and cognitive health

For both mental health and cognitive health, the results for the association with participation in voluntary work are heterogeneous (see Fig. 5e and f). Heterogeneity could not be explained by conducting subgroup analyses.

#### Social relationships

**Fig. 6:**
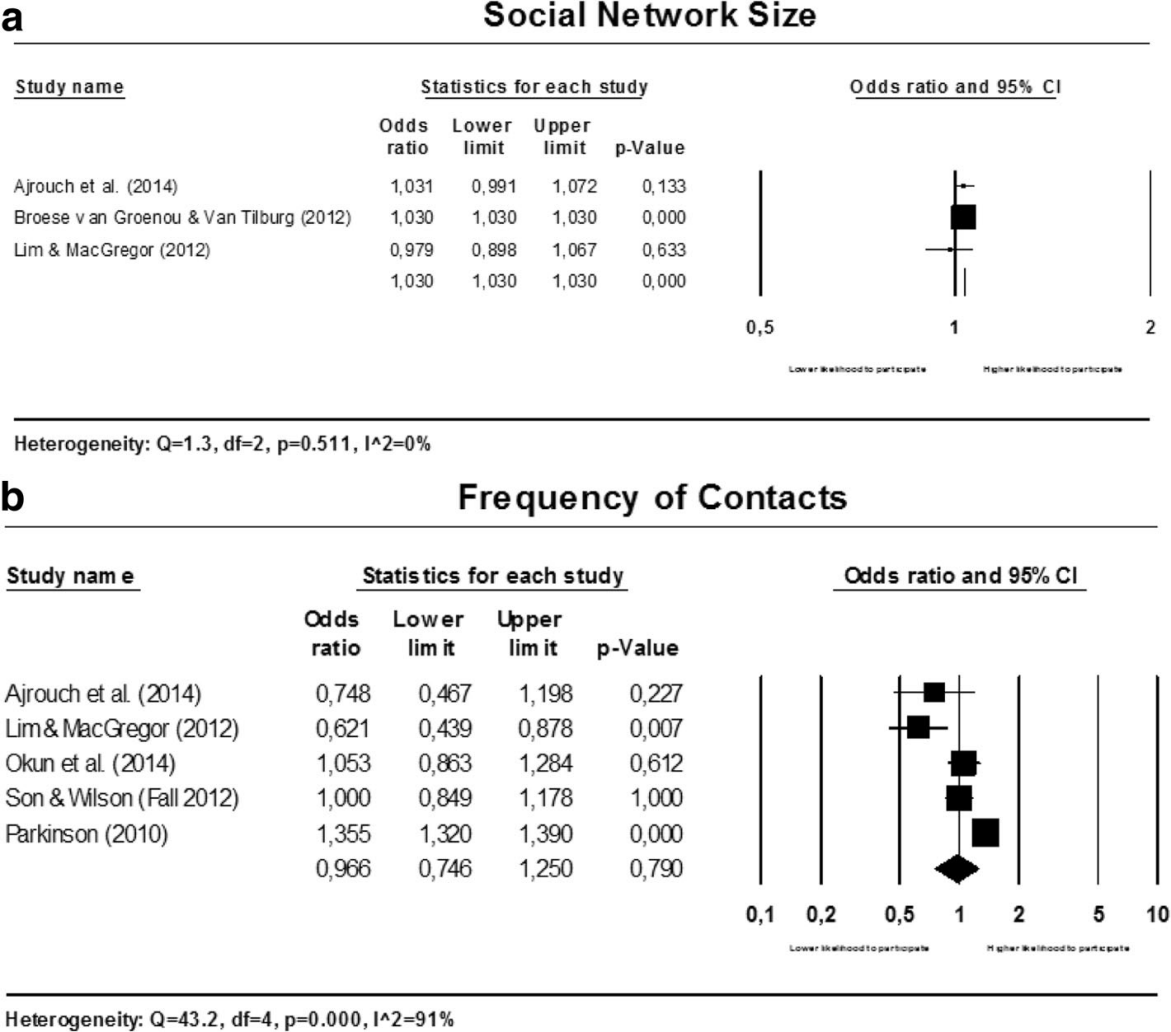
Forest plots for social relationships

The social network size and frequency of contacts are studied in relationship to participation in voluntary work. Separate meta-analyses are conducted for both factors and the forest plots are presented in multi panel Fig. 6 below.

##### Social network size

The pooled estimate shows that individuals with a larger personal social network are more likely to participate in voluntary work (OR: 1.030; 95% CI: 1.030 to 1.030) (see Fig. 6a).

##### Frequency of contacts

The results are heterogeneous and inconsistent (see Fig. 6b). Because of the large variety in the measures for frequency of social contacts used in the included studies, we did not conduct subgroup analyses to explore heterogeneity.

#### Religion

**Fig. 7:**
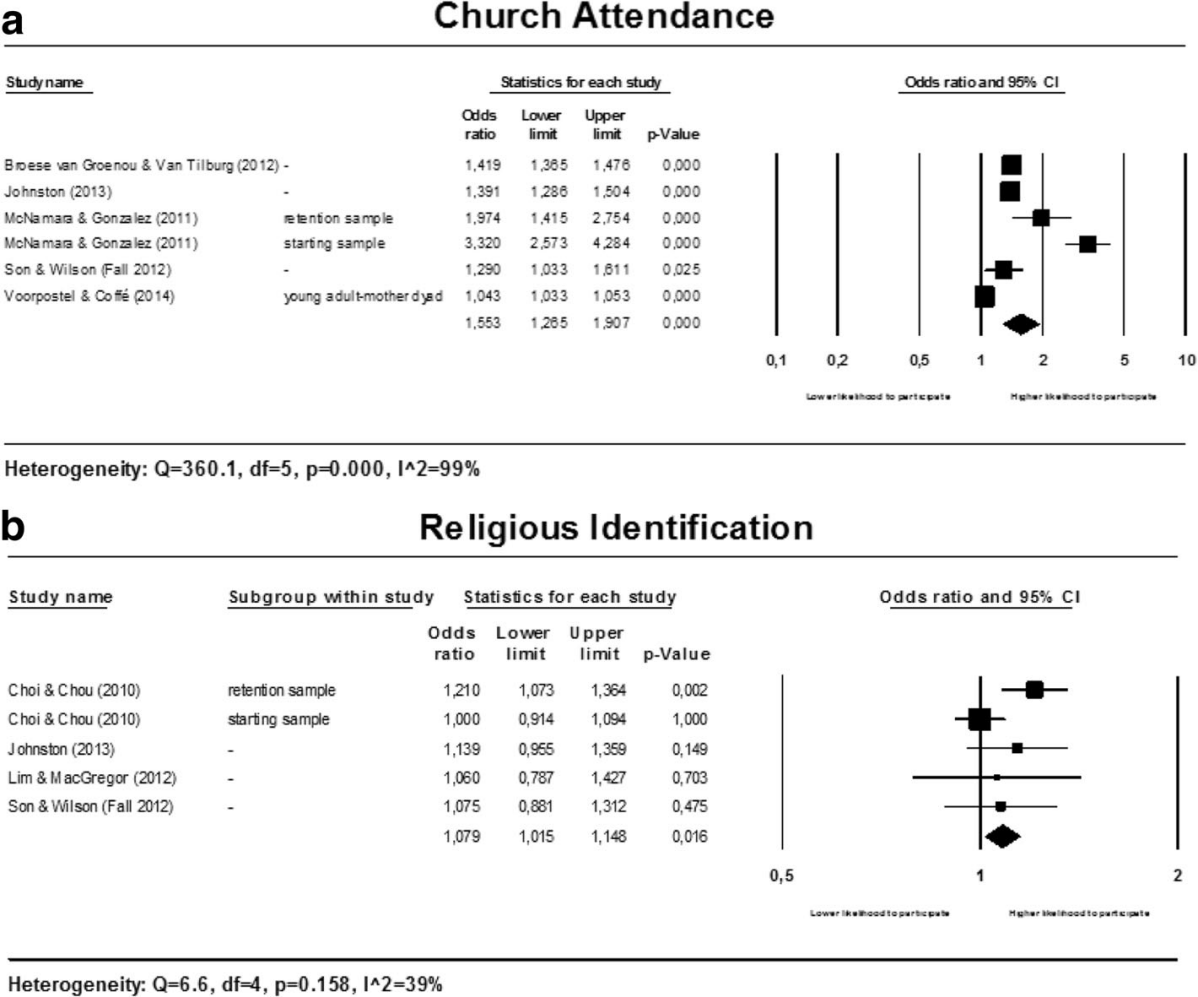
Forest plots for religion

Two factors related to religion are studied in relationship to participation in voluntary work. Meta-analyses were conducted for church attendance and religious identification. Forest plots are presented in multi panel Fig. 7 below.

##### Church attendance

The results for the association between church attendance and participation in voluntary work are heterogeneous (see Fig. 7a). Heterogeneity could not be explained by conducting subgroup analyses. However, all studies showed a positive association between church attendance and participation in voluntary work showing that church attendance and the likelihood to volunteer are positively associated. No firm conclusions can be drawn about the magnitude of the association.

##### Religious identification

The pooled estimate showed a small positive association between the level of religious identification and participation in voluntary work (OR: 1.092; 95% CI: 1.000 to 1.193) (see Fig. 7b).

#### Other factors

Two other factors are studied in relationship to participation in voluntary work. Results for the association between the frequency of attending meetings of groups, clubs and organizations (i.e. passive membership) and participation in voluntary work (i.e. active membership) are heterogeneous and inconclusive. The meta-analysis for driving status shows that people who are able to drive are more likely to participate in voluntary work. However, as the results are heterogeneous, no conclusions about the magnitude of the associations can be drawn. The forest plots are available upon request of the first author.

### Publication Bias

Publication bias was assessed for the following determinants: age, gender, marital status and educational attainment. Eggers’ test and visual inspection of the funnel plots indicate that publication bias is likely for the determinants age (Egger’s test: age (*p* = 0.007) and marital status (*p* = 0.074)). The funnel plots are available upon request.

## Discussion

This systematic review and meta-analysis aimed at identifying the contemporary determinants of participation in voluntary work. Based on the studies included in our review, we found that females (in the USA), married people and people with children (weak evidence), individuals with higher education (weak evidence) or income (especially for those individuals aged 55 and over, living in the USA and for studies in which age was taken into account as a confounder) and people who either volunteered at baseline, have a larger social network, those who are more religious and those who attend church more frequently are more likely to volunteer. In contrast, older people (weak evidence), individuals who recently had a child and individuals with a higher degree of functional limitations or increases in functional limitations are less likely to participate in voluntary work. No association with participation in voluntary work was found for employment status. There was insufficient evidence to draw firm conclusions about the association between participation in voluntary work and gender outside the USA, ethnicity, the frequency of contacts and several health related variables (overall self-rated health, cognitive health and physical health) (inconclusive results).

Many of our findings are in line with what we expected based on previous literature. First, we found that older people are less likely to volunteer. Age is believed to be related to volunteering in a curvilinear way with a peak in middle-age [9]. The studies included in our meta-analysis for age all have a mean age around middle-age or above. The mean age at baseline among the studies included in this meta-analysis varies from 42.8 years (range 25 to 74 years) [39] to 77.5 years (range 70 to 101 years) [23] and adults aged below 40 years are underrepresented in this pool of studies. Therefore, our finding that age is inversely related to participation in voluntary work confirms previous findings that showed that the likelihood to volunteer declines with age from middle-age onwards. We could not assess the association between age and volunteering before middle-age because of the inclusion of middle-aged and older adults in the studies in this meta-analysis only.

Secondly, we found no association between gender and participation in voluntary work, but we did find a positive association between being female in the USA and participation in voluntary work. Thirdly, we found that irrespective of age, married people are more likely to participate in voluntary work than unmarried people, and that this association becomes weaker with age. Besides, our analyses confirmed the importance of education and previous volunteer experiences in predicting the likelihood to volunteer. Finally, our results show that individual health status itself is not associated to participation in voluntary work, but the degree to which the individual experiences limitation in his or her functioning is. Not only the level of functional limitations was shown to be inversely associated with participation in voluntary work, also for increases in functional limitations a strong negative association with volunteering was found.

Our systematic review shows that a large number of individual factors are related to volunteering across studies and countries. Although a discussion of the theoretical links between these factors and volunteering is beyond the scope of this review, it is important to stress that many of the associations established in our meta-analysis fit into existing theoretical approaches to volunteering. For instance, Wilson and Musick (1997) in their ‘integrated theory of volunteering’ suggested that volunteering is affected by three types of capital or resources that individuals may have available: human, social and cultural resources. Many of the individual factors that were found to be associated with volunteering in our review can be clearly linked to these three types of resources. Factors like income, educational attainment and functional limitations can be viewed as indicators of the amount of human resources that individuals have available. Factors like marital status and network size constitute indicators of social resources. Finally, a factor like religiosity can be viewed as an indicator of cultural capital or resources that predispose individuals to volunteering.

### Strengths and limitations

This review was conducted according to the latest standards for conducting systematic reviews and meta-analyses. This is the first review for which findings can be generalized to the general adult population in developed countries and for which associations between identified factors and participation in voluntary work were quantified by conducting meta-analyses. We transformed all estimates into ORs in order to compare the results of included studies. A thorough overview of all determinants of volunteering studied in recent publications is provided (i.e. demographic determinants, as well as determinants related to socioeconomic status, participation in other productive activities, health status, religion and social relationships), instead of focusing on a single determinant only (e.g. health status or socioeconomic status). Updating the current state of knowledge on factors related to volunteering was important, as the research on volunteering has taken a giant leap recently. Results from our search strategy showed that compared to a decade ago, publications on factors related to volunteering have more than doubled (our search resulted in 1620 hits for the period 2000–2005 compared to 3774 hits for the period 2010–2015).

Some limitations must be mentioned as well. We limited the inclusion of studies to those published in the period 2010–2015. The choice for including this quite narrow time period was made for two main reasons. Firstly, because participation in voluntary work is related not only to individual characteristics but also to macro factors such as the demographic composition of populations, economic circumstances and government regimes, we argue that taking into account the most recent time period is the most relevant period to study in order to increase our knowledge on contemporary determinants of volunteering and provide insight in the characteristics of potential volunteers nowadays. Secondly, in trying to find a good balance between recency and efficiency, we chose to develop a rather broad search strategy without specifying any determinants beforehand, in order to provide the most comprehensive overview of all determinants studied in relation to volunteering. We cannot be sure whether our results would have been different if all studies irrespective of the date of publication were to be included. Probably the results for the factors studied in this review would be more heterogeneous due to cohort effects and probably some additional factors studied in earlier publications may have been identified. Moreover, although the studies included in this review were all published recently, the vast majority (79%) of the included studies used data with baseline measurements before the year 2005. Potentially, the results would have been different if we would have limited the inclusion of studies to those using recent data. Our search was conducted in August 2015 and several relevant articles have been published afterwards [42–46]. The results of this articles are in line with the results included in this systematic review and meta-analysis, showing for example that individuals in worse mental health are less likely to participate in voluntary work [42], religiosity in adulthood is positively associated to volunteering [43], and that previous volunteer experiences, good health and higher education are positively associated to volunteering [44]. Moreover, two studies investigated the association between providing care to grandchildren and volunteering [45, 46] but with opposite results. Therefore, it seems unlikely that including studies published after August 2015 would alter the conclusions drawn in the current study. Moreover, the inclusion of studies was limited to studies written in English, Dutch, French or German. The inclusion of only English, Dutch, French and German language studies may have led to missing some studies, however there is little evidence that exclusion of non-English-language studies leads to systematic bias in systematic reviews [47–50].

Visual inspection of the forest plots and Egger’s test have shown the presence of funnel plot asymmetry for the studies investigating the factors age and marital status in relation to the likelihood to volunteer. Therefore, these results should be interpreted with caution, as the reported effect sizes might be an overestimation of the true effect due to publication bias. However, using Egger’s approach could lead to false-positive results in the case of dichotomous outcomes [10]. However, we do not consider publication bias to be very likely. The majority of the included studies took age and marital status into account as control variables and their main interest was often directed towards the association between other factors and volunteering. Therefore, we do not expect publication bias to be a substantial problem for the results presented in this review. Funnel plot asymmetry can be caused not only by publication bias, but low methodological quality could also lead to the inflation of effects in smaller studies [10]. The latter could play a role. The majority of the studies included in this review did not provide information on the characteristics of respondents compared to participants lost to follow-up and differences between these groups could have contributed to funnel plot asymmetry.

### Recommendations for further research

The studies included in this review were very heterogeneous in terms of the methodological quality and study population. Results were also heterogeneous and, unfortunately, heterogeneity could often not be properly explained. The risk of bias assessment of the included studies has pointed out the presence of reporting flaws in included studies. Although attrition was in general high in the included studies, the majority of the studies (79.2%) did not report information regarding potential differences between participants and drop-outs, therefore insufficient information is available to assess the likelihood of attrition bias in included studies. Moreover, more than half of the studies (54.2%) did not report information on the participation rate at baseline. These are important reporting flaws, because selectivity in the study sample could have a major influence on the findings. Finally, half of the studies (50.0%) did not report information on how missing data was dealt with. For correct interpretation of the findings, it is important to know whether and how data has been imputed. In future research, more attention should be directed towards the quality of reporting as recommended in the STROBE guidelines (39).

The focus of the current systematic review and meta-analysis was to investigate the individual determinants of volunteering. Other determinants play a role as well in predicting volunteering. Contextual factors, for example, are important determinants of volunteering too [51]. Anheier & Salomon [52] (page 43) described that volunteering is determined by the way how societies are organized, how they allocate social responsibilities, and how much engagement and participation they expect from citizens. The heterogeneity between the results of the included studies could also be attributable to contextual differences between countries or cultures. Therefore, in future cross-national research on individual determinants of volunteering, it would be interesting to take into account cultural and country specific aspects. Moreover, our review showed that most studies are concentrated in the USA and selected European countries. It would be important for future research to diversify studies in terms of geographical spread. Our review has provided evidence for the association between several factors (e.g. socioeconomic status, marital status, parental status, functional health, previous volunteering, social network size and religion) and participation in voluntary work. In future research on determinants of participation in voluntary work, these factors should thus be taken into account as potential confounders in the analyses. This review has identified several gaps in the literature as well. Firstly, (weak) evidence was found for the negative association between age and volunteering from middle-age onwards. Studies including adults below middle-age were substantially underrepresented in this review. Therefore, more research should be done to examine the determinants of participation in voluntary work among younger adults and more specific, on the association between age and volunteering in younger age groups.

Secondly, more research is needed on the association between socioeconomic status and volunteering. Socioeconomic status seems to be related to the likelihood to volunteer; weak evidence for the association between education and volunteering was found and, although the overall result for the association between income and volunteering was inconclusive, for specific groups of individuals (aged 55 and over and those living in the USA) we did find a positive association. Another related factor is employment status. We did not find an association between employment status and volunteering but the pooled estimate was boundary significant and indicates the presence of a possible negative association between employment status and the likelihood to volunteer. Studies assessing the association of participation in voluntary work with employment status, level of income and education were very heterogeneous with respect to the confounders they took into account. Further research should investigate the association between these factors and participation in voluntary work, taking both educational attainment, employment status and income level into account as not only these factors themselves but also the interplay between these factors may be important in predicting participation in voluntary work.

Thirdly, this review has shown the importance of two types of life course transitions in predicting the likelihood to volunteer. For both increases in the degree to which an individual is functionally limited as well as the recent birth of a child in the household, a strong negative association with participation in voluntary work was found. Regarding parenthood, the recent birth of a child is negatively associated to volunteering whereas the presence of children in the household in general seems to be positively associated to volunteering, which shows the importance of disentangling these factors. Despite the evident importance of life course transitions in predicting the likelihood to volunteer, the majority of studies included in this review did not take them into account. In future research, the effect of major life course transition with respect to family life (for example changes in household composition, partnership status and health of family members), work (for example starting a career after graduation, transitions into and out of unemployment, changes in working hours and retirement) and health should be taken into account.

New research in the field of volunteering should aim at filling the gaps mentioned above, because volunteering is an increasingly important activity for developed societies facing aging populations. Therefore, it is important to know for policy makers which characteristics are related to volunteering in order to identify potential volunteers.

## Conclusions

In the current study, important key factors have been identified. The results of this study show that socioeconomic status, being married, social network size, church attendance and previous volunteer experiences are positively associated with volunteering and that age, functional limitations and transitions into parenthood were found to be inversely related to volunteering. A need exists for studies directed towards deepening the knowledge on the associations several between the factors and participation in voluntary work, among which are age, education, income and employment. Moreover, major life course transitions should be studied in relation to volunteering.

## Appendix 1

### Search strings MEDLINE, PsychINFO, SocINDEX, Business Source Premier and EconLit

**MEDLINE**

(“Volunteers”[MeSH:NoExp] OR “Hospital Volunteers” [MeSH].

OR

“volunteering”[tiab] OR “volunteerism”[tiab] OR “voluntary worker”[tiab] OR “voluntary workers”[tiab] OR “voluntary work”[tiab] OR “voluntary association”[tiab] OR “voluntary associations”[tiab] OR “voluntary activities”[tiab] OR “lay worker”[tiab] OR “lay workers”[tiab] OR “unpaid work”[tiab])

**AND**

(“Age Factors”[MeSH] OR “Aspirations(psychology)”[MeSH] OR “Attitude”[MeSH:NoExp] OR “Behavior” [MeSH:NoExp] OR “Causality”[MeSH:NoExp] OR “Comorbidity”[MeSH] OR “Goals”[MeSH] OR “Helping Behavior” [MeSH] OR “Intention”[MeSH] OR “Motivation”[MeSH:NoExp] OR “Psychology”[MeSH] OR “Reward”[MeSH] OR “Self Efficacy”[MeSH] OR “Sex Factors”[MeSH] OR “Sociological Factors”[MeSH].

OR

“barrier”[tiab] OR “barriers”[tiab] OR “choice”[tiab] OR “choices”[tiab]OR “characteristic”[tiab] OR “characteristics”[tiab] OR “determinant”[tiab] OR “determinants”[tiab] OR “factor”[tiab] OR “factors”[tiab] OR “goal”[tiab] OR “goals”[tiab] OR “incentive”[tiab] OR “incentives”[tiab] OR “likely”[tiab] OR “likelihood”[tiab] OR “motivation”[tiab] OR “motivations”[tiab] OR “motive”[tiab] OR “motives”[tiab] OR “predict”[tiab] OR “predicts”[tiab] OR “prediction”[tiab] OR “predictor”[tiab] OR “predictors”[tiab] OR “reason”[tiab] OR “reasons”[tiab] OR “relation”[tiab] OR “relations”[tiab] OR “relationship”[tiab] OR “why”[tiab] OR “willingness”[tiab]).

**PsychINFO**

(DE “Volunteers”.

OR

TI (volunteering OR volunteerism OR “voluntary work*”OR “volunteer work*” OR “voluntary association*” OR “unpaid work*”).

OR

AB (volunteering OR volunteerism OR “voluntary work*”OR “volunteer work*” OR “voluntary association*” OR “lay work*” OR “unpaid work*”))

**AND**

(DE “Academic Achievement Motivation” OR DE “Achievement Motivation” OR DE “Altruism” OR DE “Aspirations” OR DE “Attitudes” OR DE “Attribution” OR DE “Causal Analysis” OR DE “Commitment” OR DE “Employee Motivation” OR DE “Extrinsic Motivation” OR DE “Goals” OR DE “Human Capital” OR DE “Incentives” OR DE “Intention” OR DE “Intrinsic Motivation” OR DE “Motivation” OR DE “Needs” OR DE “Occupational Aspirations” OR DE “Organizational Commitment” OR DE “Planned Behavior” OR DE “Prediction” OR DE “Prosocial Behavior” OR DE “Reasoned Action” OR DE “Rewards” OR DE “Self Expansion” OR DE “Social Capital” OR DE “Social Behavior” OR DE “Social Perception”.

OR

TI (barrier OR barriers OR choice OR choices OR characteristic OR characteristics OR determinant OR determinants OR factor OR factors OR goal OR goals OR incentive OR incentives OR likely OR likelihood OR motivation OR motivations OR motive OR motives OR predict OR prediction OR predictor OR predictors OR reason OR reasons OR relation OR relations OR relationship OR relationships OR why OR willingness).

OR

AB (barrier OR barriers OR choice OR choices OR characteristic OR characteristics OR determinant OR determinants OR factor OR factors OR goal OR goals OR incentive OR incentives OR likely OR likelihood OR motivation OR motivations OR motive OR motives OR predict OR prediction OR predictor OR predictors OR reason OR reasons OR relation OR relations OR relationship OR relationships OR why OR willingness)).

**SocINDEX**

(DE “LAY Ministry” OR DE “VOLUNTEERS” OR DE “STUDENT volunteers in social services” OR DE “VOLUNTEER workers in social services” OR DE “VOLUNTEER service” OR DE “WOMEN volunteers in social services” OR DE “YOUNG volunteers in social services” OR DE “OLDER volunteers in social services”.

OR

TI (volunteering OR volunteerism OR “voluntary work*”OR “volunteer work*” OR “voluntary association*” OR “unpaid work*”).

OR

AB (volunteering OR volunteerism OR “voluntary work*”OR “volunteer work*” OR “voluntary association*” OR “lay work*” OR “unpaid work*”))

**AND**

(DE “ASSOCIATIONS, institutions, etc.” OR DE “ATTITUDE (psychology)” OR DE “BEHAVIORISM (psychology)” OR DE “BEHAVIOR” OR DE “HUMAN behavior” OR DE “PROSOCIAL behavior” OR DE “HELPING behavior” OR DE “PLANNED behavior theory” OR DE “MOTIVATION (psychology)” OR DE “EMPLOYEE motivation” OR DE “GOAL (psychology)” OR DE “REWARD (psychology)” OR DE “SELF-actualization (psychology)” OR DE “SELF-determination theory” OR DE “SOCIOEMOTIONAL selectivity theory” OR DE “COMMITMENT” OR DE “PSYCHOLOGY” OR DE “INFLUENCE (Psychology)” OR DE “HUMAN capital” OR DE “SOCIAL capital (Sociology)” OR DE “CULTURAL capital”.

OR

TI (barrier OR barriers OR choice OR choices OR characteristic OR characteristics OR determinant OR determinants OR factor OR factors OR goal OR goals OR incentive OR incentives OR likely OR likelihood OR motivation OR motivations OR motive OR motives OR predict OR prediction OR predictor OR predictors OR reason OR reasons OR relation OR relations OR relationship OR relationships OR why OR willingness).

OR

AB (barrier OR barriers OR choice OR choices OR characteristic OR characteristics OR determinant OR determinants OR factor OR factors OR goal OR goals OR incentive OR incentives OR likely OR likelihood OR motivation OR motivations OR motive OR motives OR predict OR prediction OR predictor OR predictors OR reason OR reasons OR relation OR relations OR relationship OR relationships OR why OR willingness).

**Business Source Premier**

DE “STUDENT volunteers in social services” OR DE “VOLUNTEERS” OR DE “VOLUNTEERS -- psychology” OR DE “VOLUNTEER recruitment” OR DE “VOLUNTEER service” OR DE “VOLUNTEER workers in income tax return preparation” OR DE “VOLUNTEER workers in social services” OR DE “WOMEN volunteers in social services” OR DE “YOUNG volunteers in social services”.

OR

TI (volunteering OR volunteerism OR “voluntary work*”OR “volunteer work*” OR “voluntary association*” OR “unpaid work*”).

OR

AB (volunteering OR volunteerism OR “voluntary work*”OR “volunteer work*” OR “voluntary association*” OR “lay work*” OR “unpaid work*”)

**AND**

DE “ATTITUDE (psychology)” OR DE “BEHAVIOR” OR DE “BEHAVIORAL research” OR DE “CONSUMER behavior” OR DE “DECISION making” OR DE “DISCRIMINATION in employment” OR DE “GENDER role in the work environment” OR DE “HUMAN behavior” OR DE “INCENTIVES in industry” OR DE “PROSOCIAL behavior” OR DE “HELPING behavior” OR DE “PLANNED behavior theory” OR DE “MOTIVATION (psychology)” OR DE “EMPLOYEE motivation” OR DE “REWARD (psychology)” OR DE “SELF-actualization (psychology)” OR DE “SELF-determination theory” OR DE “COMMITMENT” OR DE “HUMAN capital” OR DE “SOCIAL capital” OR DE “CULTURAL capital”.

OR

TI (barrier OR barriers OR choice OR choices OR characteristic OR characteristics OR determinant OR determinants OR factor OR factors OR goal OR goals OR incentive OR incentives OR likely OR likelihood OR motivation OR motivations OR motive OR motives OR predict OR prediction OR predictor OR predictors OR reason OR reasons OR relation OR relations OR relationship OR relationships OR why OR willingness).

OR

AB (barrier OR barriers OR choice OR choices OR characteristic OR characteristics OR determinant OR determinants OR factor OR factors OR goal OR goals OR incentive OR incentives OR likely OR likelihood OR motivation OR motivations OR motive OR motives OR predict OR prediction OR predictor OR predictors OR reason OR reasons OR relation OR relations OR relationship OR relationships OR why OR willingness).

**EconLit**

TI (volunteer OR volunteers OR volunteering OR volunteerism OR “voluntary work*”OR “volunteer work*” OR “voluntary association*”).

OR

AB (volunteer OR volunteers OR volunteering OR volunteerism OR “voluntary work*”OR “volunteer work*” OR “voluntary association*”)

**AND**

TI (attitude OR attitudes OR barrier OR barriers OR behavior OR choice OR choices OR characteristic OR characteristics OR commitment OR “cultural capital” OR determinant OR determinants OR factor OR factors OR goal OR goals OR “human capital” OR incentive OR incentives OR likely OR likelihood OR motivation OR motivations OR motive OR motives OR predict OR prediction OR predictor OR predictors OR reason OR reasons OR relation OR relations OR rewards OR relationship OR relationships OR “social capital” OR why OR willingness).

OR

AB (attitude OR attitudes OR barrier OR barriers OR behavior OR choice OR choices OR characteristic OR characteristics OR commitment OR “cultural capital” OR determinant OR determinants OR factor OR factors OR goal OR goals OR “human capital” OR incentive OR incentives OR likely OR likelihood OR motivation OR motivations OR motive OR motives OR predict OR prediction OR predictor OR predictors OR reason OR reasons OR relation OR relations OR rewards OR relationship OR relationships OR “social capital” OR why OR willingness)

## Appendix 2

**Table 7:** Risk of Bias assessment tool (based on QUIPS, Hayden et al. [13])

Domain	Items
1. Study participation	1a. Method used to identify population: recruitment of participants for the study was performed in a consecutive way 1b. Adequate study participation: at least 70% of recruited individuals agreed to participate
2. Study attrition	2a. Adequate follow-up rate: at least 80% of the baseline study participants participated at follow-up 2b. There are no important differences between participants who completed the study and those who did not
3. Determinant measurement	3a. Adequate proportion of complete data: at least 70% of the study sample has complete data on the determinant(s) 3b. The method and setting of determinant measurement is the same for all study participants 3c. Appropriate methods of imputation are used for missing determinant data
4. Outcome measurement	4a. Outcome measure truly captures participation in voluntary work and does not allow for participation in informal caregiving or other productive activities not equal to volunteering, unless subgroups are made for the distinct forms of participation 4b. The method and setting of outcome measurement is the same for all study participants
5. Study confounding	5a. The following potentially important confounders are measured: a1. age a2. socioeconomic status (e.g. education, income) a3. gender a4. participation in voluntary work at baseline 5b. The method and setting of measurement of the confounders is the same for all study participants 5c. Appropriate methods of imputation are used for missing data regarding the confounders 5d. The following potentially important confounders are accounted for in the study design (e.g., matching for key variables, stratification, or initial assembly of comparable groups) or in the analysis (i.e., appropriate adjustment) d1. age d2. socioeconomic status (e.g. education, income) d3. gender d4. participation in voluntary work at baseline
6. Statistical analysis and reporting	6a. The selected statistical model is adequate for the design of the study design 6b. There is no over fitting (at least 10 participants in the smallest group per determinant and outcome variable) 6c. There is no selective reporting of results

## Appendix 3

**Table 8:** Determinants measured in included studies

Author	Determinants^a^
Ajrouch et al. [18]	Social network (size, proportion of family, age, proximity, frequency), education, age, gender, race, health limitation and depression
Bartels et al. [19]	Ratio government expenditure / GDP, interest in politics, children, education, marital status, income, liking the neighbourhood
Bekkers [20]	Trust
Broese van Groenou & Van Tilburg [21]	Age, gender, cohort, education, employment status, health status, marital status, size of personal network and church attendance
Choi & Chou [22]	Education, income, health, work status, religion, generative qualities, number of meetings attended, age, marital status, ethnicity and gender
Cramm & Nieboer [23]	Age, gender, ethnicity, marital status, education, social capital, social functioning, cognitive functioning and physical functioning, volunteering at baseline
Curl et al. [24]	Driving status, waves since driving cessation, gender, age, ethnicity, education, marital status, household income, depressive symptoms, chronic conditions, self-rated health, IADL limitations and cognitive ability
Curl et al. [25]	For both the individual and the spouse: driving status, waves since driving cessation, age, ethnicity, education, couple income, cognitive ability, chronic conditions, IADL limitations and self-rated health
Einolf & Philbrick [26]	Marriage (covariates taken into account but no effect size provided are: volunteering at baseline, ethnicity, education, age, health, hours worked, religious attendance, housework hours and children
Hank & Erlinghagen [27]	Gender, age, education, partnership status, employment status, self-rated health, country
Johnston [28]	Religious importance, religious attendance, family income, functional health, employment status, child currently at home, marital status
Lim & Mac Gregor [29]	Age, gender, ethnicity, income, education, marriage, children, social involvement index, voluntary group involvement, religious tradition, religious index, region, volunteering at baseline, number of close friends, ethnicity of friends, religiosity of friends
McNamara & Gonzales [30]	Age, ethnicity, gender, volunteering at baseline, assets, education, income, health, marital status, volunteer status of spouse, like to spend time with spouse, spousal caregiving, parental caregiving, children, employment status, provision of informal help in community, religious attendance
Mike et al. [31]	Age, gender, education, personality traits: conscientiousness, extraversion, agreeableness, neuroticism and openness, current work status
Nesbit [32]	Volunteering at baseline, gender, age, ethnicity, education, birth of child, divorce, death in family
Okun et al. [33]	Volunteer satisfaction and enjoyment, age, gender, race, hours worked p/wk., education, functional limitations, social interaction, attending clubs /organizations and church attendance
Parkinson [34]	Area of residence, country of birth, English proficiency, education, health care insurance, living arrangements, transport, SF36, DSSI, number of visits to healthcare professionals
Pavlova & Silbereisen [35]	Coping strategies for occupational uncertainty, region, community size, gender, education, income, employment status, partnership status and general health
Pavlova & Silbereisen [36]	Perceived activation demands, volunteering at baseline, age and self-rated health
Son & Wilson [37]	Generativity,religious identification, church attendance, spirituality, religious coping, parental religion, parental sociability, education, age, gender, ethnicity, marital status and income
Son & Wilson [38]	Altruistic obligation, civic obligation, religious identification, spirituality, religious coping, public religiosity, parental religion, education, age, gender, ethnicity, marital status, income, employment, physical health, religious tradition, contact frequency with friends
Son & Wilson [39]	Hedonic well-being, eudemonic well-being, social well-being, age, gender, ethnicity, marital status, education, income, employment, church attendance, physical health
Voorpostel & Coffé [40]	Transitions in partnership, transitions in parental status, age, (change in) education, (change in) employment status, volunteering at baseline
Voorpostel & Coffé [41]	Parental separation, parental levels of voting and volunteering, parental occupation and education, young adults living situation, age, gender, schooling, education, occupation and church visits

^a^The determinants listed here are only those determinants for which the association with the outcome is measured longitudinally and are therefore eligible for inclusion in the meta-analysis

## Appendix 4

**Table 9:** Overview of determinants and studies included in meta-analyses

Determinant	Articles that reported an association between the determinant and the outcome	Studies selected for inclusion in meta-analysis
Demographic Variables		
Age	Ajrouch et al. (16); Broese van Groenou en Van Tilburg (11); Choi & Chou (19); Cramm & Nieboer (20); Curl et al. (21); Curl et al. (22); Hank & Erlinghagen (24); Lim & MacGregor (26); McNamara & Gonzales (27); Mike et al. (28); Nesbit (29); Okun et al. (30); Pavlova & Silbereisen (33); Son & Wilson (34); Son & Wilson (35); Son & Wilson (36); Voorpostel & Coffé (37); Voorpostel & Coffé (38)	Ajrouch et al. (16); Broese van Groenou en Van Tilburg (11); Cramm & Nieboer (20); Curl et al. (21); Hank & Erlinghagen (24); Lim & MacGregor (26); Nesbit (29); Okun et al. (30); Pavlova & Silbereisen (33); Son & Wilson (36); Voorpostel & Coffé (37)
Gender	Ajrouch et al. (16); Broese van Groenou en Van Tilburg (11); Choi & Chou (19); Cramm & Nieboer (20); Curl et al. (21); Hank & Erlinghagen (24); Lim & MacGregor (26); McNamara & Gonzales (27); Mike et al. (28); Nesbit (29); Okun et al. (30); Pavlova & Silbereisen (32); Son & Wilson (34); Son & Wilson (35); Son & Wilson (36); Voorpostel & Coffé (38)	Ajrouch et al. (16); Broese van Groenou en Van Tilburg (11); Cramm & Nieboer (20); Curl et al. (21); Hank & Erlinghagen (24); Lim & MacGregor (26); Nesbit (29); Okun et al. (30); Pavlova & Silbereisen (32); Son & Wilson (36); Voorpostel & Coffé (38)
Ethnicity	Ajrouch et al. (16); Choi & Chou (19); Cramm & Nieboer (20); Curl et al. (21); Curl et al. (22); Lim & MacGregor (26); McNamara & Gonzales (27); Nesbit (29); Okun et al. (30); Parkinson (31); Son & Wilson (34); Son & Wilson (35); Son & Wilson (36)	Ajrouch et al. (16); Cramm & Nieboer (20); Curl et al. (21); Lim & MacGregor (26); Nesbit (29); Okun et al. (30); Parkinson (31); Son & Wilson (36)
Marital Status	Bartels et al. (17); Broese van Groenou en Van Tilburg (11); Choi & Chou (19); Cramm & Nieboer (20); Curl et al. (21); Einolf & Philbrick (23); Hank & Erlinghagen (24); Johnston (25); Lim & MacGregor (26); McNamara & Gonzales (27); Pavlova & Silbereisen (32); Son & Wilson (34); Son & Wilson (35); Son & Wilson (36); Voorpostel & Coffé (37)	Bartels et al. (17)^a^; Broese van Groenou en Van Tilburg (11); Cramm & Nieboer (20); Curl et al. (21); Hank & Erlinghagen (24); Johnston (25); Lim & MacGregor (26); Pavlova & Silbereisen (32); Son & Wilson (36); Voorpostel & Coffé (37)
Parental Status	Bartels et al. (17); Johnston (25); Lim & MacGregor (26); McNamara & Gonzales (27); Nesbit (29); Voorpostel & Coffé (37)	Bartels et al. (17)^29^; Johnston (25); Lim & MacGregor (26); McNamara & Gonzales (27); Nesbit (29); Voorpostel & Coffé (37)
Socioeconomic Status		
Educational Attainment	Ajrouch et al. (16); Bartels et al. (2013); Broese van Groenou en Van Tilburg (11); Choi & Chou (19); Cramm & Nieboer (20); Curl et al. (21); Curl et al. (22); Hank & Erlinghagen (24); Lim & MacGregor (26); McNamara & Gonzales (27); Mike et al. (28); Nesbit (29); Okun et al. (30); Parkinson (2010); Pavlova & Silbereisen (32); Son & Wilson (34); Son & Wilson (35); Son & Wilson (36); Voorpostel & Coffé (37); Voorpostel & Coffé (38)	Ajrouch et al. (16); Bartels et al. (2013)^29^; Broese van Groenou en Van Tilburg (11); Cramm & Nieboer (20); Curl et al. (21); Hank & Erlinghagen (24); Lim & MacGregor (26); Nesbit (29); Okun et al. (30); Parkinson (2010); Pavlova & Silbereisen (32); Son & Wilson (36); Voorpostel & Coffé (38)
Income	Bartels et al. (2013); Choi & Chou (19); Curl et al. (21); Curl et al. (22); Johnston (25); Lim & MacGregor (26); McNamara & Gonzales (27); Pavlova & Silbereisen (32); Son & Wilson (34); Son & Wilson (35); Son & Wilson (36)	Bartels et al. (2013)^29^; Curl et al. (21); Johnston (25); Lim & MacGregor (26); Pavlova & Silbereisen (32); Son & Wilson (36)
Participation in productive activities		
Volunteering at Baseline	Cramm & Nieboer (20); Lim & MacGregor (26); McNamara & Gonzales (27); Nesbit (29); Pavlova & Silbereisen (33); Son & Wilson (36); Voorpostel & Coffé (37)	Cramm & Nieboer (20); Lim & MacGregor (26); McNamara & Gonzales (27); Nesbit (29); Pavlova & Silbereisen (33); Son & Wilson (36); Voorpostel & Coffé (37)
Employment Status	Broese van Groenou en Van Tilburg (11); Choi & Chou (19); Hank & Erlinghagen (24); Johnston (25); McNamara & Gonzales (27); Okun et al. (30); Pavlova & Silbereisen (32); Son & Wilson (35); Son & Wilson (36)	Broese van Groenou en Van Tilburg (11); Hank & Erlinghagen (24); Johnston (25); McNamara & Gonzales (27); Pavlova & Silbereisen (32); Son & Wilson (36)
Health status		
Overall health Status	Choi & Chou (19); Curl et al. (21); Curl et al. (22); McNamara & Gonzales (27); Hank & Erlinghagen (24); Pavlova & Silbereisen (32); Pavlova & Silbereisen (33); Son & Wilson (36)	Curl et al. (21); Hank & Erlinghagen (24); Pavlova & Silbereisen (32); Son & Wilson (36)
Limitations in ADL	Ajrouch et al. (16); Broese van Groenou en Van Tilburg (11); Curl et al. (21); Curl et al. (22); Johnston (25); Okun et al. (30)	Ajrouch et al. (16); Broese van Groenou en Van Tilburg (11); Curl et al. (21); Johnston (25)
Physical Health	Cramm & Nieboer (20); Curl et al. (21); Curl et al. (22); Parkinson (31)	Cramm & Nieboer (20); Curl et al. (21); Parkinson (31)
Mental Health	Ajrouch et al. (16); Curl et al. (21); Parkinson (31)	Ajrouch et al. (16); Curl et al. (21); Parkinson (31)
Cognitive Health	Cramm & Nieboer (20); Curl et al. (21); Curl et al. (22)	Cramm & Nieboer (20); Curl et al. (21)
Social relationships		
Social Network Size	Ajrouch et al. (16); Broese van Groenou en Van Tilburg (11); Lim & MacGregor (26)	Ajrouch et al. (16); Broese van Groenou en Van Tilburg (11); Lim & MacGregor (26)
Frequency of Contacts	Ajrouch et al. (16); Lim & MacGregor (26); Okun et al. (30); Parkinson (31); Son & Wilson (36)	Ajrouch et al. (16); Lim & MacGregor (26); Okun et al. (30); Parkinson (31); Son & Wilson (36)
Religion		
Church Attendance	Broese van Groenou en Van Tilburg (11); Johnston (25); McNamara & Gonzales (27); Okun et al. (30); Son & Wilson (36); Voorpostel & Coffé (38)	Broese van Groenou en Van Tilburg (11); Johnston (25); McNamara & Gonzales (27); Son & Wilson (36); Voorpostel & Coffé (38)
Religious Identification	Choi & Chou (19); Johnston (25); Lim & MacGregor (26); Son & Wilson (36)	Choi & Chou (19); Johnston (25); Lim & MacGregor (26); Son & Wilson (36)
Other		
Driving Status	Curl et al. (21); Parkinson (31)	Curl et al. (21); Parkinson (31)
Attending Meetings	Choi & Chou (19); Okun et al. (30)	Choi & Chou (19); Okun et al. (30)

^a^Bartels et al. (2013) present several models. The results from the Panel Data Logit with Fixed Effects (XtLogit FE) model were used in our analyses

## Abbreviations

CIs: Confidence Intervals
ORs: Odds Ratios
PRISMA: Preferred Reporting Items for Systematic Reviews and Meta-Analyses
QUIPS: Quality In Prognosis Studies
USA: United States of America
